# Characterization and comparison of recombinant full‐length ursine and human sex hormone‐binding globulin

**DOI:** 10.1002/2211-5463.13341

**Published:** 2021-12-13

**Authors:** Anne Mette Frøbert, Malene Brohus, Julia N. C. Toews, Phillip Round, Ole Fröbert, Geoffrey L. Hammond, Michael T. Overgaard

**Affiliations:** ^1^ Department of Chemistry and Bioscience Faculty of Engineering and Science Aalborg University Denmark; ^2^ Department of Cellular & Physiological Sciences The University of British Columbia Vancouver BC Canada; ^3^ Department of Cardiology Faculty of Health Örebro University Sweden; ^4^ Department of Clinical Medicine Faculty of Health Aarhus University Denmark; ^5^ Department of Clinical Pharmacology Aarhus University Hospital Denmark; ^6^ Steno Diabetes Center Aarhus Aarhus University Hospital Denmark

**Keywords:** hibernation, insect cells, sex hormone‐binding globulin, steroid, *Ursus arctos*

## Abstract

Sex hormone‐binding globulin (SHBG) regulates the bioavailability of sex steroid hormones in the blood. Levels of SHBG increase markedly in brown bears (*Ursus arctos*) during hibernation, suggesting that a key regulatory role of this protein is to quench sex steroid bioavailability in hibernation physiology. To enable characterization of ursine SHBG and a cross species comparison, we established an insect cell‐based expression system for recombinant full‐length ursine and human SHBG. Compared with human SHBG, we observed markedly lower secretion levels of ursine SHBG, resulting in a 10‐fold difference in purified protein yield. Both human and ursine recombinant SHBG appeared as dimeric proteins in solution, with a single unfolding temperature of ~ 58 °C. The thermal stability of ursine and human SHBG increased 5.4 and 9.5 °C, respectively, in the presence of dihydrotestosterone (DHT), suggesting a difference in affinity. The dissociation constants for [^3^H]DHT were determined to 0.21 ± 0.04 nm for human and 1.32 ± 0.10 nm for ursine SHBG, confirming a lower affinity of ursine SHBG. A similarly reduced affinity, determined from competitive steroid binding, was observed for most steroids. Overall, we found that ursine SHBG had similar characteristics to human SHBG, specifically, being a homodimeric glycoprotein capable of binding steroids with high affinity. Therefore, ursine SHBG likely has similar biological functions to those known for human SHBG. The determined properties of ursine SHBG will contribute to elucidating its potential regulatory role in hibernation physiology.

## Graphical abstract

We established an insect cell‐based expression system to characterize recombinant ursine SHBG. Ursine SHBG appeared to have two signal peptide cleavage sites, was dimeric in solution, and had a single unfolding temperature which increased in the presence of dihydrotestosterone. Determination of dissociation constants for several steroids revealed that ursine SHBG binds steroids with lower affinity than human SHBG.

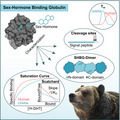

**Abbreviations**
AEX: anion exchange chromatography
DCC: dextran‐coated charcoal
DHT: 5α‐dihydrotestosterone
MALDI‐ToF MS: matrix‐assisted laser desorption ionization‐time of flight mass spectrometry
pFBD: pFastBac™ Dual
RBA: relative binding affinity
SEC‐MALS: size exclusion chromatography‐multi‐angle light scattering
SHBG: sex hormone‐binding globulin

Sex hormone‐binding globulin (SHBG) is a plasma protein that transports and regulates the bioavailability of sex steroids in the blood by adjusting the equilibrium between free and protein‐bound steroid [1]. Steroids are lipophilic with low solubility in water and are therefore bound to various binding proteins when transported in the circulation [2]. Sex steroids, including androgens and estrogens, are important regulatory molecules that control sexual maturation and reproduction as well as regulate other sexually dimorphic tissues like muscle, adipose tissue, and bone [3]. According to the ‘free hormone hypothesis’, only free steroids can enter target cells by passive diffusion through the plasma membrane and exert their functions [4].

The plasma level of SHBG increases 45‐fold in Scandinavian brown bears during hibernation [5]. During the 6–month‐long hibernation period, the bear does not eat, drink, urinate, or defecate, and moves only infrequently [6]. In a period where energy conservation is critical, such an increase in SHBG suggests an unknown function of this protein during this important physiological state in bears. Contrary to humans, brown bears do not show any signs of disease from prolonged immobility and obesity [7, 8, 9]. High plasma SHBG levels in humans correlate with a lower risk of numerous diseases, such as metabolic syndrome, type 2 diabetes, cardiovascular diseases [10], and muscle weakness [11]—none of which are observed in the hibernating brown bear [7]. Elucidating the physiological mechanisms that protect the hibernating bear may reveal novel therapeutic targets in humans.

Human SHBG is well characterized [12, 13, 14, 15, 16, 17, 18]. Like the human SHBG precursor polypeptide, the ursine SHBG precursor polypeptide is 402 amino acids long, and they share 77% sequence identity. Cleavage of the human 29 amino acid secretion signal peptide results in mature human SHBG of 373 amino acids [19], but the signal peptide cleavage site and mature sequence has not been determined for ursine SHBG prior to this study. Mature human SHBG has a deglycosylated molecular weight of 40.5 kDa and forms a homodimer with a total glycosylated weight of about 90 kDa [19, 20, 21]. Each monomer consists of two laminin G‐like domains (LG domains), joined in tandem by a short linker. The tertiary structure of the N‐terminal LG domain has been solved for human SHBG, and the steroid‐binding site is located close to the center of this domain in a hydrophobic core formed between two β‐sheets (Fig. 1A) [22]. Additionally, a yet uncharacterized receptor, located in the plasma membrane of sex steroid‐responsive cells, has been reported for human SHBG [23, 24]. Amino acid residues 48–57 (TWDPEGVIFY) in the N‐terminal LG domain of human SHBG were identified as the receptor‐binding site (residues 51–60 in ursine SHBG) (Fig. 1B) and this region is highly conserved between species [25].

**Fig. 1:**
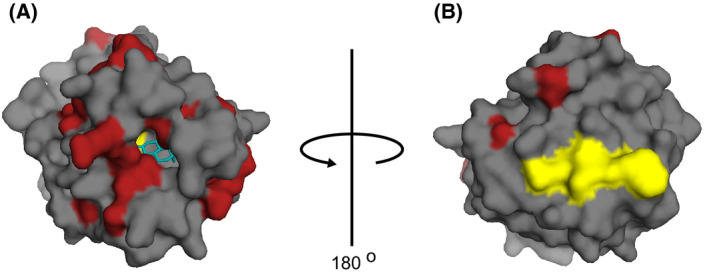
The human SHBG N‐terminal LG domain. (A) Front and (B) back view of the human SHBG N‐terminal LG domain (surface representation) with DHT marked with blue and the receptor binding site marked with yellow (PDB: 1D2S) [22]. Substitutions from human to ursine SHBG are marked with red (note that only substitutions positioned at the surface are visible). The structure in figure B has been rotated 180° about the *y*‐axis from figure A.

To characterize ursine SHBG, we established an insect cell‐based expression system for recombinant ursine SHBG alongside the human orthologue. The expression system produced dimeric and active full‐length ursine and human SHBG. A lower steroid‐binding affinity of ursine SHBG, compared to human, was observed. Except for the lower steroid affinity, ursine SHBG showed similar characteristics to human SHBG, and therefore, they likely have similar biological functions. The determined properties of ursine SHBG will contribute to elucidate SHBG’s potential regulatory role in hibernation physiology.

## Results and Discussion

### Sequence alignment and homology modeling of ursine SHBG

**Fig. 2:**
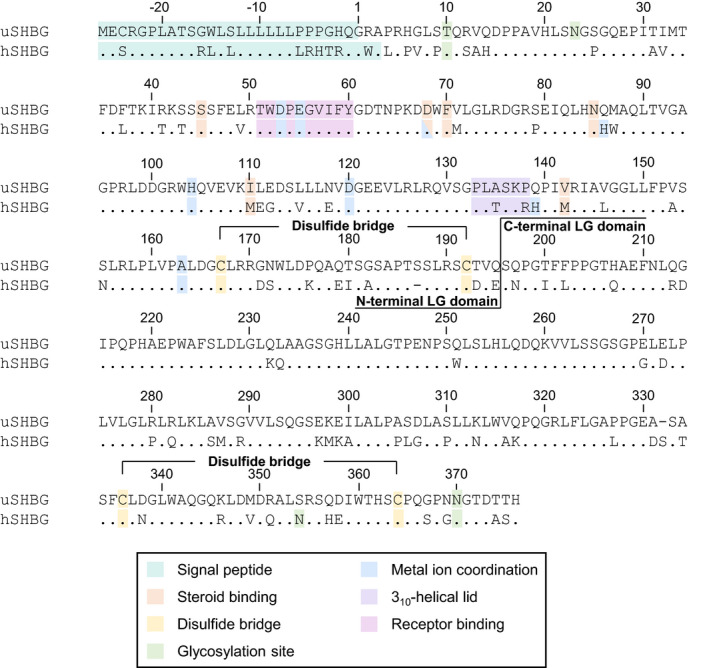
Alignment of the human and ursine SHBG amino acid sequences. The Homo sapiens SHBG (hSHBG) (NCBI: NP_001031) and Ursus arctos horribilis SHBG (uSHBG) (NCBI: XP_026376673) sequences were aligned by clustal omega (version 1.2.4, European Bioinformatics Institute, University College Dublin, Dublin, Ireland). Conserved residues are marked with a period in the human sequence. The sequences are numbered according to the mature ursine sequence.

**Fig. 3:**
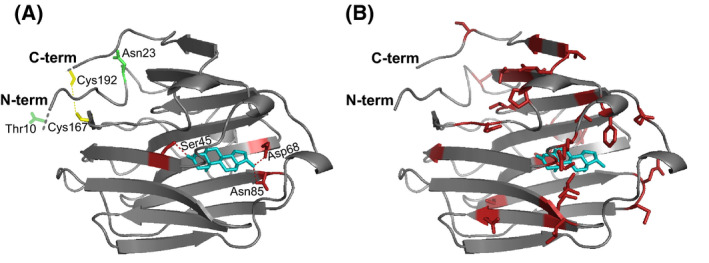
Homology model of the ursine SHBG N‐terminal LG domain. (A) The structure of the ursine SHBG N‐terminal LG domain was modeled using the SWISS‐MODEL server and the structure of the human orthologue in complex with dihydrotestosterone (DHT) as template (PMDB: PM0084113). Glycosylation sites are shown in green and the disulfide bond in yellow. The structure has been extended in the N‐ and C‐terminal ends to include Thr10 and Cys192 (shown in dashes). (B) The modeled ursine SHBG N‐terminal LG domain with amino acids differing from human SHBG shown as red sticks.

The ursine SHBG amino acid sequence was aligned to that of human SHBG (Fig. 2). The structure of the ursine SHBG N‐terminal LG domain was predicted by homology modeling based on the structure of the human orthologue (Fig. 3). In human SHBG, steroids are anchored in the steroid‐binding site through hydrogen bonds, with androgens and estrogens binding in opposite orientations. The C3 carbonyl of androgens and the C17 hydroxyl of estrogens points into the interior of SHBG and forms a hydrogen bond with the side‐chain hydroxyl of Ser42 in human SHBG, which is of paramount importance for high‐affinity steroid binding. Two additional hydrogen bonds are formed at the entrance of the binding pocket between the hydroxyl group at C17 of androgens or C3 of estrogens and the side chains of Asp65 and Asn82 in human SHBG, where Asp65 appears to be the most important [22, 26]. These three steroid‐anchoring residues are conserved in ursine SHBG (Ser45, Asp68, Asn85) (Table 1). Besides these residues, all other contacts are hydrophobic, with Phe67, Met107, and Met139 in human SHBG contributing with the largest contact surface to the steroid [26]. Phe67 is conserved in ursine SHBG (Phe70), while the two Met residues are substituted to Ile (Ile110) and Val (Val142). Trp84 in human SHBG also affects steroid‐binding, although not by direct contact [26], and is substituted to Met (Met87) in ursine SHBG.

**Table 1:** Conservation of residues in ursine SHBG with known functional importance in human SHBG. The SHBG sequences are numbered starting from the first amino acid of the mature sequence, where the signal peptide of ursine SHBG is cleaved between position 26 and 27. For the N‐linked glycosylation sites, the entire consensus sequence is stated with the residue, to which the glycosylation is attached, marked in bold.

Interaction	Amino acid in human SHBG	Amino acid in ursine SHBG	Conserved
Steroid‐binding	Ser42	Ser45	Yes
	Asp65	Asp68	Yes
	Phe67	Phe70	Yes
	Asn82	Asn85	Yes
	Met107	Ile110	No
	Met139	Val142	No
3_10_‐helical lid	130–135 (PLTSKR)	133–138 (PLASKP)	Partly
R_SHBG_ binding site	48–57 (TWDPEGVIFY)	51–60 (TWDPEGVIFY)	Yes
Disulfide bridges	Cys164‐Cys188, Cys333‐Cys361	Cys167‐Cys192, Cys336‐Cys364	Yes
*O*‐linked glycosylation	Thr7	Thr10	Yes
*N*‐linked glycosylation		**Asn23**‐Gly‐Ser	No
	**Asn351**‐Arg‐Ser	**Ser354**‐Arg‐Ser	No
	**Asn367**‐Gly‐Thr	**Asn370**‐Gly‐Thr	Yes
Ca^2+^‐binding site I	Asp50, Glu52, Ala160	Asp53, Glu55, A163	Yes
Zn^2+^‐binding site II	Asp65	Asp68	Yes
	His83	Gln86	No
	His136	Gln139	No
Zn^2+^‐binding site III	His101, Asp117	His104, Asp120	Yes

SHBG forms two disulfide bridges (Cys164‐Cys188 and Cys333‐Cys361 in human SHBG), which are highly conserved across species [19]. The four cysteines are also conserved in ursine SHBG and are therefore expected to link Cys167 to Cys192 at the very C terminus of the N‐terminal LG domain (Fig. 3) and Cys336 to Cys364 in the C‐terminal LG domain.

Human SHBG is a glycoprotein with one *O*‐linked (Thr7) and two *N*‐linked glycosylation sites (Asn351 and Asn367) [27, 28]. The *O*‐glycosylation site is fully occupied, while the Asn351 and Asn367 *N*‐glycosylation sites have average molar occupancies of 85% and 95%, respectively [27]. The *O*‐linked glycosylation site is conserved in ursine SHBG (Thr10). The consensus site for *N*‐glycosylation Asn367‐Gly‐Thr (Asn‐X‐Ser/Thr, where X is not a Pro) is also conserved in ursine SHBG (Asn370‐Gly‐Thr), as in most species [29], while Asn351 is substituted to Ser in ursine SHBG (Ser354). However, Pro22 in the human orthologue is substituted with Ser in ursine SHBG (Ser25), giving rise to a new consensus site for ursine *N*‐glycosylation: Asn23‐Gly‐Ser (Fig. 2). This site is located at the surface of SHBG and is therefore potentially accessible for glycosylation (Fig. 3). The degree of occupancy of these three glycosylation sites in ursine SHBG is not known.

Three bivalent metal ion coordination sites have been identified in the N‐terminal LG domain of human SHBG: A Ca^2+^ binding site located opposite of the steroid‐binding pocket, a Zn^2+^ binding site located at the entrance to the steroid‐binding pocket, and another Zn^2+^‐binding site located close to the dimer interface. The Ca^2+^‐binding site and the Zn^2+^‐binding site at the dimer interface are conserved in ursine SHBG. For the Zn^2+^‐binding site located at the entrance to the steroid‐binding site, two of the human Zn^2+^‐coordinating residues, His83 and His136, are substituted to Gln in the bear (Gln86 and Gln139, respectively). Therefore, this Zn^2+^‐binding site is most likely absent in ursine SHBG. In human SHBG, this Zn^2+^‐binding site is located close to a flexible 3_10_‐helical loop (residues 130–135 in human SHBG) that covers the entrance of the steroid‐binding pocket when Zn^2+^ is absent. Zn^2+^ coordination causes a structural change of the loop that decreases the SHBG‐binding affinity for estrogens, while this has little or no effect on androgens [30, 31]. One of the residues in this flexible lid is substituted in ursine SHBG (Thr132 to Ala135).

### Expression of full‐length SHBG in ExpiSf cells

To characterize the biochemical properties of SHBG, we established an expression and purification protocol for recombinant ursine and human protein variants. SHBG, fused to a C‐terminal FLAG purification tag, was expressed in ExpiSf insect cells from a baculovirus expression vector system. The ExpiSf cell line produced higher SHBG yields compared to test expressions using Sf9 or High Five insect cells or human embryonic kidney (HEK293) cells (data not shown). Maximal levels of SHBG in the expression medium were observed 72–96 h postinfection. However, as increasing amounts of degraded protein was apparent over the same period of time (data not shown), SHBG was harvested 72 h postinfection.

**Fig. 4:**
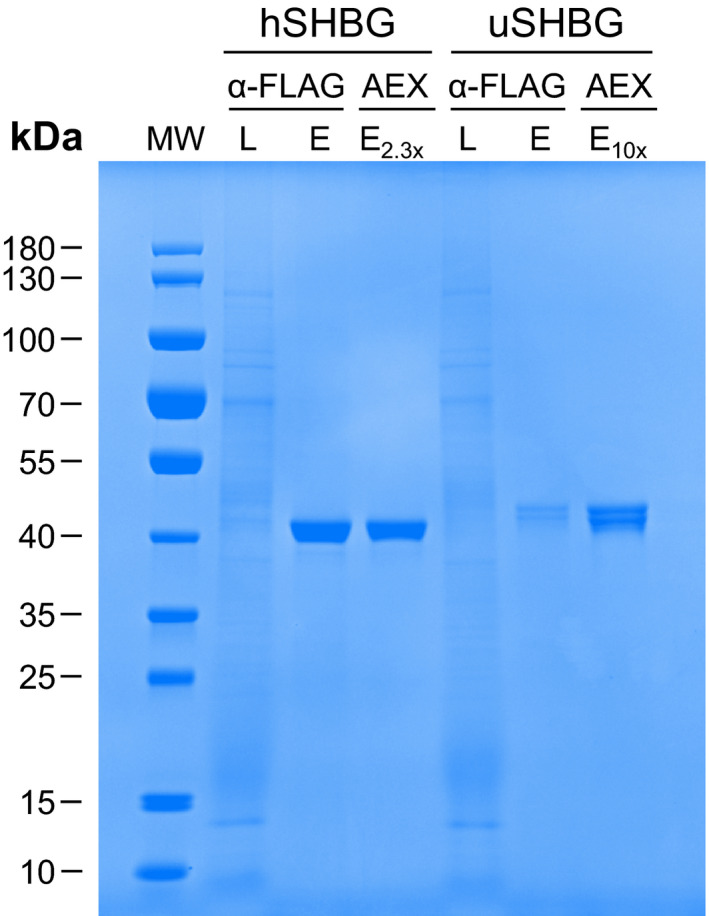
Efficiency of SHBG purification. SDS/PAGE analysis (Coomassie Brilliant Blue R‐250 stained) showing the efficiency of anti‐FLAG affinity chromatography (α‐FLAG) and anion exchange chromatography (AEX) for purification of ursine (uSHBG) and human SHBG (hSHBG) from the ExpiSf expression medium. L = load, E = eluate. Equal volumes are loaded of both human and ursine SHBG; however, the AEX eluate were concentrated 2.3× for human SHBG and 10× for ursine SHBG.

Supplementation of 10 µm DHT to the expression medium increased the SHBG yield from ExpiSf cells by 68% for ursine SHBG and 20% for human SHBG (Fig. S1). Based on preliminary analysis on SHBG expressed in insect cells, we speculate that this might be due to an increased solubility of SHBG upon steroid binding and that the relative effect on increase in solubility is larger for ursine compared to human SHBG (data not shown). However, there might be other explanations, and the effect of adding steroid is not fully understood. The SHBG fusion protein was purified to > 99% by using a combination of anti‐FLAG affinity and anion exchange chromatography (AEX), removing all protein contaminants visible by SDS/PAGE with Coomassie R‐250 staining (Fig. 4). MALDI‐ToF mass spectrometry (MS) confirmed the identity of SHBG, with 89.3% sequence coverage for ursine SHBG and 96.3% for human SHBG (Fig. S2). The final yield (mean ± SD of three replicates) of purified SHBG per liter ExpiSf culture was 65.9 ± 7.5 µg (1.6 ± 0.2 nmol) for ursine SHBG and about 10‐fold higher for human SHBG (653 ± 133 µg, 15.8 ± 3.2 nmol).

### Ursine and human SHBG contains two signal peptide cleavage sites

**Fig. 5:**
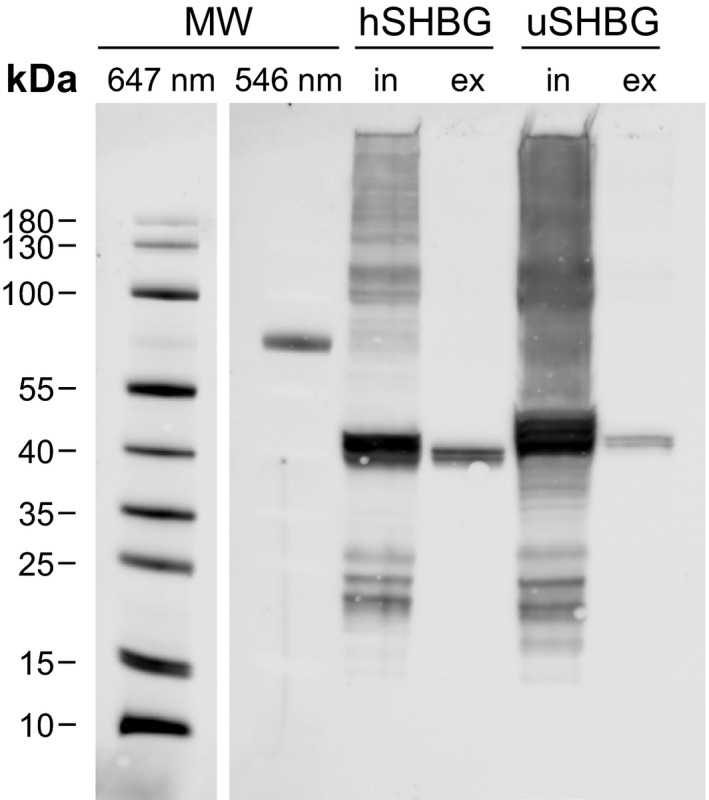
Intra‐ and extracellular SHBG. Nonreducing western blot of intracellular (in) and extracellular (ex) ursine (uSHBG) and human SHBG (hSHBG) expressed by ExpiSf cells. Comparable amounts are loaded of cells (intracellular) and expression medium (extracellular)—thus, the cells were resuspended to the original volume of expression medium. Ursine samples are loaded in a 3× higher volume than the human.

Cleavage of the signal peptide was evident from western blotting analysis where the apparent molecular weight of SHBG in the conditioned medium was slightly lower than that of SHBG from the cell pellet (Fig. 5). We therefore concluded that SHBG present in the medium had passed through the secretory pathway. Because SHBG is a plasma protein and thus natively secreted from hepatocytes, it is important that the recombinant protein enters the secretory pathway in the host cell to be properly processed, including attachment of carbohydrates and disulfide bond formation [32].

MALDI‐ToF MS revealed no peaks corresponding to tryptic or chymotryptic peptides from the signal peptides (human: MESRGPLATSRLLLLLLLLLLRHTRQGWA(L), ursine: MECRGPLATSGWLSLLLLLLPPPGHQ(G)), indicating that the secretion signal peptides had been cleaved off SHBG. A peak corresponding to the most N‐terminal semichymotryptic peptide from mature human SHBG (LRPVLPTQSAHDPPAVHLSNGPGQEPIAVMTF) confirmed cleavage of the signal peptide at the same site as reported previously [33]. We also observed a peak corresponding to an N‐terminal semichymotryptic peptide with an alternative cleavage site (RPVLPTQSAHDPPAVHL) for human SHBG. This alternative N‐terminal has previously been reported with an occurrence of ~ 25% [33]. For ursine SHBG, two peaks with masses corresponding to N‐terminal semichymotryptic peptides with two different signal peptide cleavage sites were apparent, namely between residues 26 and 27 (GRAPRHGL) and between residues 27 and 28 (RAPRHGL) (Fig. S3). Both cleavage sites are predicted as the most likely by the SignalP algorithm, although this depends on the software version used [34].

### Substitution of the secretion signal peptide does not increase SHBG yield

**Fig. 6:**
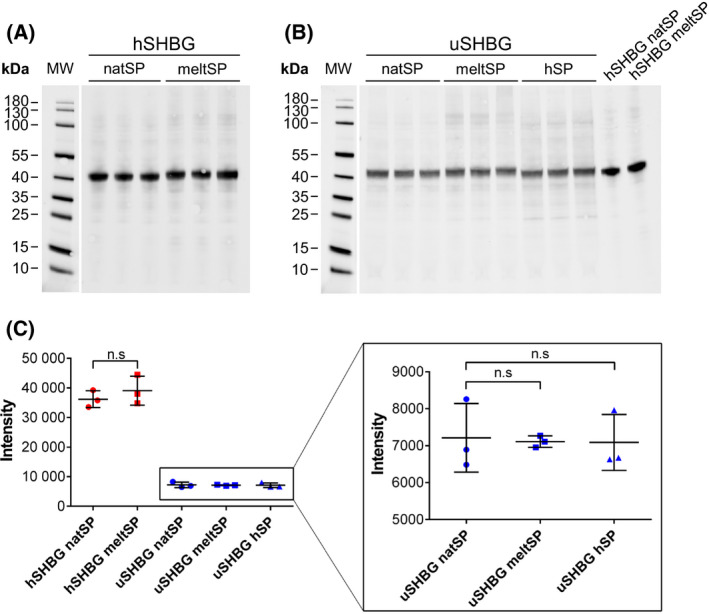
Substitution of the SHBG signal peptide. Western blot of triplicate ExpiSf cultures expressing human (hSHBG) and ursine SHBG (uSHBG) with native (natSP), melittin (meltSP), or human SHBG signal peptide (hSP). (A) 10 μL culture medium with human SHBG was loaded. (B) 20 μL culture medium with ursine SHBG was loaded along with 5 μL culture medium with human SHBG used to normalize the quantities. (C) imagej was used to quantify the protein bands on the western blots. The intensities have been adjusted with the dilution factors. Means and standard deviations are indicated. The band intensities were not significantly different (n.s) (*P*‐value ≥ 0.05) between the various signal peptides, as determined from an unpaired Student’s *t*‐test.

A large fraction of both human and ursine SHBG was retained within the ExpiSf cells compared with the amount secreted to the expression medium (Fig. 5). Furthermore, the amount of ursine SHBG secreted to the expression medium was lower than that of human SHBG, despite similar total expression levels (intra‐ and extracellular) and similar cell densities. This indicates that ursine SHBG is less efficiently secreted from the ExpiSf cells (Fig. 5). In an attempt to optimize secretion efficiency of both ursine and human SHBG from the ExpiSf cells, the native signal peptides were substituted with the signal peptide of honeybee melittin, which has been reported to increase the yield of other proteins from insect cells [35]. Additionally, the signal peptide of ursine SHBG was substituted with the human SHBG signal peptide. Theoretically, the ursine SHBG signal peptide (MECRGPLATSGWL SLLLLLLPPPGHQ(G)) is less efficient than the human (MESRGPLATSRLLLLLLLLLLRHTRQGWA), as it contains a shorter hydrophobic core, fewer polar amino acids in the C‐terminal, and several proline residues close to the cleavage site [36, 37]. A longer leucine stretch, such as the one found in the human SHBG signal peptide, has been reported to increase the yield of hyenid SHBG in CHO cells [38]. However, substitution of the ursine signal peptide to that of human SHBG did not significantly increase secretion of ursine SHBG from ExpiSf insect cells. Neither did substitution of either the human or ursine signal peptide to that of honeybee melittin (Fig. 6). Thus, an inefficient signal peptide cannot explain the observed low yield of secreted ursine SHBG from the ExpiSf cells.

### Recombinant ursine and human SHBG expressed in ExpiSf cells is dimeric

**Fig. 7:**
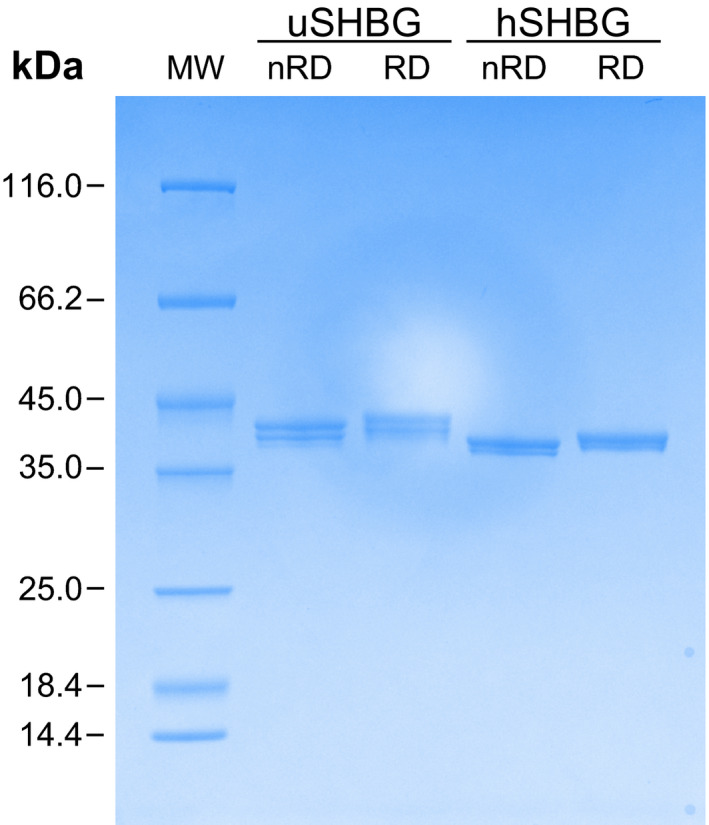
Reduced and nonreduced SHBG. SDS/PAGE analysis of nonreduced (nRD) and reduced (RD) purified ursine (uSHBG) and human SHBG (hSHBG).

SDS/PAGE analysis of reduced and nonreduced purified recombinant SHBG showed a small change in migration, supporting formation of disulfide bonds in both the ursine and human protein variants (Fig. 7). The presence of the Cys164‐Cys188 disulfide bond in human SHBG was verified directly by MALDI‐ToF MS of tryptically digested SHBG under non‐reducing conditions (Fig. S4). The tryptic dipeptide containing the Cys333‐Cys361 disulfide bond in human SHBG, and the dipeptide containing the expected Cys336‐Cys364 disulfide bond in ursine SHBG were not observed. These dipeptides both include *N*‐linked glycosylation sites, which may explain their lack of detection. Also, the tryptic dipeptide harboring the predicted ursine SHBG Cys167‐Cys192 disulfide was not detected. This is likely due to the relatively large mass (8690.40 Da), which is known to reduce the efficiency of ionization and detection in MALDI‐ToF.

**Fig. 8:**
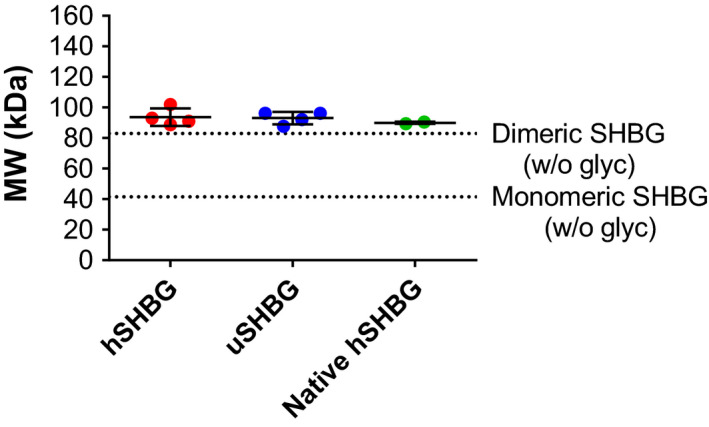
Determination of SHBG molecular mass by SEC‐MALS. The masses of human and ursine recombinant SHBG and of native human serum SHBG, determined by SEC‐MALS. Means and standard deviations are indicated. Lines corresponding to the molecular weight of monomeric and dimeric SHBG (nonglycosylated) is shown.

In native PAGE, recombinant ursine and human SHBG migrated a similar distance as SHBG present in human serum, suggesting identical multimeric composition (Fig. S5). Indeed, size exclusion chromatography‐multiangle light scattering (SEC‐MALS) analysis verified the mass of both recombinant and native serum SHBG to be ~ 90 kDa, demonstrating that these proteins form dimers in solution (Fig. 8; Fig. S6).

### Ursine SHBG has three potential glycosylation sites

Ursine SHBG contains the same number of glycosylation consensus sites as the human orthologue, although positioned differently. The masses of SHBG, determined from SEC‐MALS analysis (93.1 ± 4.1 for ursine SHBG and 93.7 ± 5.8 kDa for human) demonstrate that the recombinant SHBG is glycosylated, as these masses are larger than the theoretical nonglycosylated dimeric masses (83.1 kDa for ursine SHBG and 82.9 kDa for human). The mass differences correspond to ~ 29 and ~ 32 sugar units per SHBG‐monomer for ursine and human SHBG, respectively. Three bands from ursine SHBG and two bands from human SHBG were observed in SDS/PAGE analysis (Fig. 7) and were expected to derive from differential glycan occupancy, as previously described for human plasma SHBG [27]. Moreover, both ursine and human SHBG were observed to elute in more than one peak in AEX chromatography suggesting that various glycoforms occur (data not shown). The masses of recombinant intact monomeric SHBG were determined by MALDI‐ToF MS to 43.1 kDa for ursine SHBG and 43.5 kDa for human SHBG (Fig. S7). This corresponds to a mass shift of 1.55 kDa for ursine SHBG and 2.05 kDa for human SHBG, or approximately 9 and 12 sugar units, respectively, relative to the theoretical molecular weight determined from the amino acid sequence (Table 2). Additionally, the peak from human SHBG contained a shoulder, corresponding to a mass of 42.8 kDa and a mass shift of 1.34 kDa or 8 sugar units. Thus, the glycosylations of recombinant human SHBG expressed in insect cells are either fewer or smaller than those of native human SHBG, for which the *O*‐glycosylation typically is 3 sugar units and the two *N*‐glycosylations typically are 11 units each [27]. Since the native glycosylations of SHBG are not associated with steroid‐binding, protein folding, or dimerization, we do not believe that the insect‐derived glycosylations affect any of the experimental outcomes in this study [12].

**Table 2:** SHBG masses determined by MALDI‐ToF MS. Masses of intact human and ursine recombinant SHBG (with a C‐terminal FLAG‐tag) determined by MALDI‐ToF MS and calculated from the mature amino acid sequence. For ursine SHBG, the calculated mass of the mature protein, cleaved after both amino acid 26 and 27 in the preprotein, is stated.

	[SHBG+H]^+^	[SHBG+2H]^2+^	[SHBG+3H]^3+^	[SHBG]
	Measured *m*/*z*			
Human SHBG	43 534.87	21 760.10	14 487.84	
Ursine SHBG	43 073.48	21 575.08	NOB	
	Calculated *m*/*z*			Calculated average mass (Da)
Human SHBG	41 460.11	20 730.56	13 820.71	41 459.10
Ursine SHBG (384aa)	41 566.01	20 783.51	13 856.01	41 565.00
Ursine SHBG (383aa)	41 508.96	20 754.98	13 836.99	41 507.95
	δ (Da)			
Human SHBG	2074.77	1029.54	667.13	
Ursine SHBG (384aa)	1507.47	791.58	NOB	
Ursine SHBG (383aa)	1564.52	820.10	NOB	
	Measured mass (Da)			Measured mass (Da) (mean ± SD)
Human SHBG	43 533.86	43 516.17	43 454.46	43 501.50 ± 41.69
Ursine SHBG	43 072.47	43 146.14	NOB	43 109.30 ± 52.09
				Mass shift (Da)
Human SHBG				2042.40
Ursine SHBG (384aa)				1544.31
Ursine SHBG (383aa)				1601.36

The *N*‐glycosylation consensus site positioned closest to the C terminus of SHBG (Asn367‐Gly‐Thr in human SHBG) is highly conserved in all known mammalian sequences, including the bear, which suggests that glycosylation of this site in particular is functionally important [29]. We were unable to identify a peak corresponding to the nonglycosylated peptide spanning this *N*‐glycosylation site in ursine SHBG (Asn370), neither in MALDI‐ToF analysis of tryptic peptides of our recombinantly expressed SHBG, nor in the data from the bear plasma proteomics study by Welinder et al. [5]. This suggests high glycan occupancy of this site in both native and recombinant ursine SHBG, consistent with the observations for native human SHBG, which has a reported glycan occupancy of 95% [27]. Glycan occupancy results in a mass shift of the peptide, and the resulting modified peptide can only be identified by MALDI‐ToF MS if the glycan mass is known.

A peak corresponding to the nonglycosylated peptide spanning the *O*‐glycosylation site in ursine SHBG (Thr10) was observed by MALDI‐ToF analysis of recombinant SHBG but not in the proteomics data by Welinder et al. Thus, for recombinant ursine SHBG, the *O*‐glycosylation site is not, or only partially, glycosylated, while it seems to be fully occupied in native ursine SHBG. The *O*‐glycosylation site has been reported to be fully occupied for native human SHBG [27].

A peak corresponding to the nonglycosylated peptide covering the *N*‐glycosylation site Asn23 found in ursine SHBG, but not in human SHBG, was observed both for the recombinant SHBG and in the proteomics data by Welinder et al. [5]. We therefore suggest that this site is not, or only partially, glycosylated. Based on these data, we believe that the size heterogeneity of ursine plasma SHBG observed by Welinder et al. [5] derives primarily from partial occupation of the Asn23 glycosylation site.

### Ursine and human recombinant SHBG is active and stabilized by steroid‐binding

**Fig. 9:**
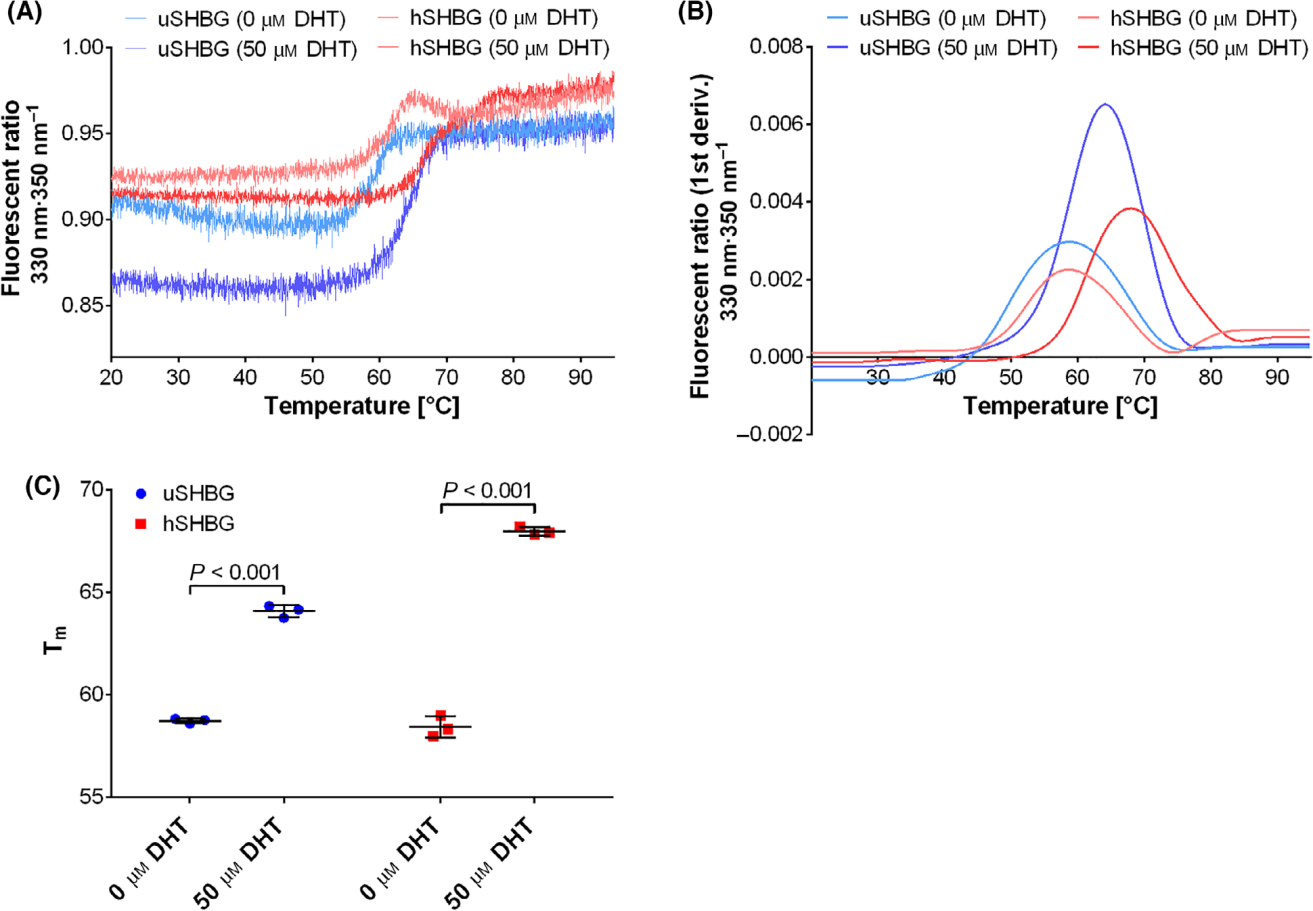
Thermal unfolding assay of SHBG with and without DHT. Thermal unfolding assay with (A) fluorescence ratio 350/330 nm and (B) first derivative hereof as a function of temperature for ursine (uSHBG) and human SHBG (hSHBG) in the presence of 0 or 50 µm DHT. (C) Unfolding temperature (*T*_m_) of ursine (uSHBG) and human SHBG (hSHBG) in the presence of 0 or 50 µm DHT determined from the peak maximum of the first derivative of the fluorescent ratio 330/350 nm as a function of the temperature. Means and standard deviations are indicated. *P*‐values were determined from an unpaired Student’s *t*‐test.

The ability of the recombinant SHBG to bind steroid was demonstrated by thermal unfolding analysis in the presence and absence of DHT. Supplementation of 50 µm DHT increased the unfolding temperature from 58.7 ± 0.1 °C to 64.1 ± 0.3 °C for ursine SHBG and from 58.4 ± 0.5 °C to 68.0 ± 0.2 °C for human SHBG (Fig. 9). Thus, DHT‐binding stabilized both ursine and human SHBG, as has previously been reported for human SHBG [31]. Interestingly, human SHBG is stabilized to a larger extend by DHT than ursine SHBG (a 9.6 °C versus a 5.4 °C increase in unfolding temperature, respectively), suggesting a higher DHT‐binding affinity of the human variant.

### Ursine SHBG binds steroids with a 6‐fold lower affinity than human SHBG

We then determined the steroid‐binding affinity of recombinant ursine and human SHBG by Scatchard analysis using [^3^H]DHT as the labeled ligand [39].

**Fig. 10:**
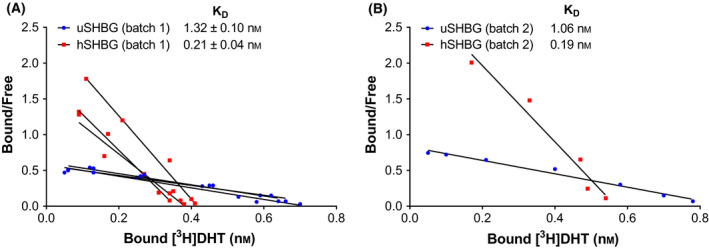
Scatchard analysis of DHT binding to SHBG. Scatchard analysis used to determine the steroid‐binding affinity of purified recombinant ursine SHBG (uSHBG) to [^3^H]DHT compared to human SHBG (hSHBG). (A) The analysis was performed in three technical replicates for the first recombinant SHBG batch and (B) once for the replicate on a second SHBG batch. The dissociation constant (*K*_D_) was determined as the negative reciprocal of the slope of the linear regression.

From saturation curves of specifically bound [^3^H]DHT, plotted as a function of total [^3^H]DHT added, a concentration of ~ 6 nm [^3^H]DHT was determined to be sufficient to saturate the applied amount of SHBG which results in minimal nonspecific binding and economy in the use of [^3^H]DHT (Fig. S8). The dissociation constant (*K*_D_) for DHT (mean ± SD) was determined to be 1.32 ± 0.10 nm for ursine SHBG and 0.21 ± 0.04 nm for human SHBG, from triplicate Scatchard plot analysis, as the negative reciprocal of the slope (Fig. 10A). Thus, recombinant ursine SHBG bound DHT with a 6.4‐fold lower affinity than human SHBG. A single analysis on a second SHBG batch demonstrated very similar affinities (Fig. 10B). The determined *K*_D_ value for recombinant human SHBG (0.21 ± 0.04 nm) is in fair agreement to that previously reported for human serum SHBG (0.4 nm) [40].

Next, the SHBG binding affinities for 3β‐androstanediol, testosterone, androstenediol, and estradiol were assessed using [^3^H]DHT as the labeled ligand in a conventional competitive steroid‐binding assay. Half maximal inhibitory concentrations (IC_50_), that is, the steroid concentration able to outcompete 50% of the bound [^3^H]DHT, were used to determine the relative binding affinities (RBA) to DHT (Table 3). The RBA values were similar between human and ursine SHBG for these steroids, meaning that their binding affinities to ursine SHBG are also 6‐fold lower compared to human SHBG, except for 3β‐androstanediol where ursine SHBG demonstrated a slightly but significantly higher relative affinity. In addition, the RBA of both human and ursine SHBG for dehydroepiandrosterone, androstenedione, progesterone, deoxycorticosterone, 11‐deoxycortisol, and corticosterone were determined to be < 1% of the DHT affinity (data not shown). The IC_50_ values for SHBG ligands that effectively displaced DHT from the binding site (i.e., RBA > 1%) were then used to deduce their affinities (*K*_D_ values) for SHBG by using the Cheng–Prusoff equation (Table 3) [41].

**Table 3:** Half maximal inhibitory concentrations, relative binding affinities, and dissociation constants. Half maximal inhibitory concentrations (IC_50_) of [^3^H]DHT binding to SHBG of different steroids. Relative binding affinities to DHT and dissociation constants (K_D_) were calculated from the IC_50_ values.

	IC_50_ Mean ± SD		RBA (DHT = 100%) Mean ± SD		*P*‐value (*t*‐test)	K_D_ (nm) Mean ± SD	
	Human SHBG	Ursine SHBG	Human SHBG	Ursine SHBG		Human SHBG	Ursine SHBG
DHT	5.00 ± 1.10	6.14 ± 0.77	100	100	–	0.17 ± 0.03	1.18 ± 0.13
3β‐androstanediol	10.02 ± 1.83	8.83 ± 0.81	45.42 ± 8.06	66.51 ± 2.20	0.04	0.34 ± 0.04	1.78 ± 0.16
Testosterone	24.35 ± 5.32	34.48 ± 1.90	21.50 ± 0.27	18.52 ± 1.66	0.09	0.83 ± 0.16	6.41 ± 0.43
Androstenediol	57.55 ± 4.87	68.75 ± 3.78	8.40 ± 1.53	8.72 ± 0.38	0.76	1.88 ± 0.23	13.33 ± 0.44
Estradiol	110.04 ± 17.13	145.48 ± 23.57	4.71 ± 0.42	4.41 ± 0.10	0.34	3.63 ± 0.55	27.06 ± 4.52

Our *in silico* analyses of ursine SHBG showed that some of the residues involved in steroid binding differ from those of human SHBG and that substitutions occur within the steroid‐binding pocket (Fig. 1). This could explain the lower steroid‐binding affinity of ursine when compared to human SHBG. Specific amino acid substitutions, identical to those found in the ursine SHBG steroid‐binding pocket, have previously been introduced into human SHBG to determine how particular residues contribute to steroid binding. Introduction of a M107I substitution in human SHBG lowered the DHT‐binding affinity about 4‐fold with the estradiol‐binding affinity being relatively unchanged [42]. Human SHBG with a M139V substitution displayed a 2‐ to 3‐fold reduced affinity for DHT [38]. Since the affinity of ursine SHBG is ~ 6‐fold lower than human SHBG, we believe that both the M107I and the M139V substitutions contribute to this lowered affinity.

Apart from Old World primates, lower steroid affinity, relative to human SHBG, is a common feature of SHBG in other mammals, including dogs (*K*_D_ = 7.1 nm [40]) and hyenas (*K*_D_ = 1.40–1.95 nm [38]), which are both phylogenetically closer to bears than humans. However, the SHBG affinity for the different sex steroids relative to each other, namely DHT > testosterone > estradiol, is conserved between ursine and human SHBG, as in other species [31, 43], which suggests that SHBG functions similarly across mammalian species.

### Zinc binding does not affect estradiol affinity of ursine SHBG

The relative binding affinity of human and ursine SHBG for estradiol was assessed in the presence and absence of Zn^2+^. It has previously been shown that Zn^2+^ coordination has little or no effect on the affinity of human SHBG for androgens [30], and we do therefore not expect the addition of Zn^2+^ to affect the binding of [^3^H]DHT notably. The presence of 1 mm Zn^2+^ inhibited the ability of estradiol to outcompete [^3^H]DHT binding to human SHBG, while Zn^2+^ had no effect on the affinity of estradiol toward ursine SHBG (Table 4). Thus, the two ursine His to Gln substitutions in the Zn^2+^ coordination site located at the entrance to the steroid‐binding pocket eliminates the Zn^2+^‐induced reduction in affinity for estradiol observed in human SHBG [26]. The same substitution of the His136 residue has also previously been reported to disrupt the Zn^2+^‐binding site in human SHBG [30, 44]. The physiological function of the Zn^2+^‐induced reduction in estradiol affinity of human SHBG is not clear, but has been suggested to regulate the availability of sex steroids in Zn^2+^‐rich tissues, such as the prostate and other male reproductive organs [44]. Based on our results, we conclude that Zn^2+^ does not influence steroid binding of SHBG in bears, as seems to be the case in other mammals apart from humans, since residues within the loop segment covering the steroid‐binding pocket are poorly conserved between species, including those critically involved in the binding of Zn^2+^ in this location [30].

**Table 4:** Test of Zn^2+^‐induced reduction in estradiol affinity. The effect of supplementation of 1 mm Zn^2+^ on the affinity of estradiol and thus estradiol’s ability to outcompete [^3^H]DHT from the steroid‐binding site of SHBG. Estradiol was added in an amount corresponding approximately to the IC_50_ value in the absence of Zn^2+^, that is, the amount capable of outcompeting 50% of [^3^H]DHT.

Zn^2+^	Bound [^3^H]DHT (%)	
	Human SHBG	Ursine SHBG
0 mm	57.42	40.47
1 mm	90.17	38.63

### Our characterization of ursine SHBG will contribute to elucidate the role of SHBG during hibernation in bears

In conclusion, we established an insect cell‐based expression system for recombinant full‐length ursine and human SHBG. This cell system was able to produce dimeric and active full‐length ursine and human SHBG. Apart from a 6‐fold lower steroid affinity, ursine SHBG had similar characteristics to human SHBG, namely being a homodimeric glycoprotein capable of binding steroids with high affinity. Therefore, ursine SHBG likely has similar biological functions as known for human SHBG.

The previous observation of increased SHBG levels in brown bears during hibernation suggests that tight regulation of sex steroid activity is physiologically important [5]. To further delineate the importance of SHBG in bear hibernation, the plasma concentration of SHBG during hibernation and active state should be determined together with the sex steroid concentrations. These data, together with our determined *K*_D_ of SHBG for various sex steroids, would allow calculation of the free steroid levels. Such information could help determine if sex steroid activity is an important regulatory component of hibernation physiology or if SHBG has other important steroid‐free functions.

Further elucidation of the protective mechanisms that underlie the bears’ outstanding ability to avoid complications related to immobility and obesity, including the potential involvement of SHBG, may be a stepping‐stone to facilitate the development of new human clinical therapies.

## Experimental procedures

### Homology model of ursine SHBG

A homology model of the ursine SHBG N‐terminal LG domain (PMDB: PM0084113) was generated in the SWISS‐MODEL workspace [45] and visualized in pymol [46]. The model was based on the crystal structure of the N‐terminal LG domain of human SHBG in complex with DHT (PDB: 1D2S) [22]. It should be emphasized that the model of the ursine SHBG N‐terminal LG domain does not take energy optimization into account and thus only illustrates the overall structure.

### Expression of recombinant SHBG

Liver tissue from a euthanized brown bear was kindly provided by Orsa Rovdjurspark, Sweden. Ethics approval was not required for this study, since no live animals were used, and since euthanasia of the surplus animal was independently decided by Orsa Rovdjurspark. The liver tissue was homogenized by freeze drying followed by bead‐beating and the RNA extracted by using the NucleoSpin® RNA kit (MACHEREY‐NAGEL GmbH & Co. KG, Düren, Germany). Ursine and human native *SHBG* cDNA was generated and amplified by reverse transcription followed by PCR using the ursine RNA and MVP human liver total RNA (Agilent Technologies, Inc., Santa Clara, CA, USA) as template and the primers listed in Table S1. The cDNA was cloned into a pFastBac™ Dual (pFBD) plasmid (Gibco™, Invitrogen Life Technologies, Carlsbad, CA, USA) downstream to the polyhedrin promoter with a C‐terminal FLAG‐tag incorporated. The native signal peptide of human and ursine SHBG was substituted with that of honeybee melittin (MKFLVNVALVFMVVYISYIYAAPEP) and for ursine SHBG also with that of human SHBG (MESRGPLATSRLLLLLLLLLLRHTRQGWA) by PCR using the pFBD plasmids as templates, whereafter the *SHBG* constructs were cloned into pFBD as above. The pFBD plasmids were transformed into MAX Efficiency® DH10Bac™ Competent *E. coli* cells (Gibco™, Invitrogen Life Technologies) for site‐specific transposition of *SHBG* into the bacmid vector according to the method described in the user manual ‘Bac‐to‐Bac® Baculovirus Expression System’ (version D, 6 April 2004, 10359) from Invitrogen™ (Life Technologies, Carlsbad, CA, USA). Correct transposition of the *SHBG* constructs into the bacmids was verified by sequencing.

The insect cell line ExpiSf9™ (Gibco™, Invitrogen Life Technologies) was transfected and cultivated according to the manufacturer’s instructions (‘ExpiSf™ Expression System’, Publication Number MAN0017532, Revision A.0). Briefly, ExpiSf9™ cells were cultured in ExpiSf CD medium (Gibco™, Invitrogen Life Technologies) supplemented with 1% PenStrep (100 units·mL^−1^ penicillin, 100 g·mL^−1^ streptomycin) (Gibco™, Invitrogen Life Technologies) at 27 °C with normal atmosphere and 95 rpm orbital shaking (50‐mm shaking diameter). The ExpiSf9™ cells were transfected with the bacmid DNA with use of ExpiFectamine™ Sf Transfection Reagent (Gibco™, Invitrogen Life Technologies) to generate recombinant baculovirus which was further amplified and harvested when the viability dropped to 60–80%. The baculoviral titer was determined by a flow cytometric tittering assay using the Baculovirus Envelope gp64 Monoclonal Antibody (AcV1), PE, eBioscience™ (Invitrogen™, Life Technologies). Only viral stocks with titers ≥ 1 × 10^8^ ivp·mL^−1^ were applied for protein expression. For protein expression, ExpiSf cells were seeded on the day before infection to a final density of 5 × 10^6^ viable cells·mL^−1^ in fresh ExpiSf™ CD Medium and added ExpiSf™ Enhancer (Gibco™, Invitrogen Life Technologies). After 18–24 h, the cells were infected with a multiplicity of infection ≥ 5. The expression medium was harvested 72 h postinfection where cells were removed by centrifugation at 500 rcf for 15 min (RT) and remaining cell debris by centrifuging at 18 600 ***g*** for 30 min (4 °C). 1 mm CaCl_2_ and 0.02% sodium azide were added to stabilize the protein and prevent microbial growth.

### Protein purification

Ursine and human SHBG with native signal peptide was purified from 1 L cultures by anti‐FLAG affinity chromatography and anion exchange chromatography.

1 mL (settled) equilibrated Pierce™ Anti‐DYKDDDDK Affinity Resin (Pierce Biotechnology, Inc., Thermo Fisher Scientific, Rockford, IL, USA) was added per liter expression medium and incubated overnight on a roller mixer at 4 °C. The resin was collected from the expression medium by centrifugation at 1000 ***g*** for 5 min and thereafter transferred to an empty gravity flow column. The resin was washed with 100 mL binding buffer (50 mm Tris‐HCl, 150 mm NaCl, 2.5 mm CaCl_2_, pH 7.5). Twice during the wash step, the column was plugged and capped, and the column mixed over end with buffer, before eluting.

FLAG‐tagged SHBG was eluted from the resin by 30 min incubation with 2 mL FLAG elution buffer [binding buffer supplemented with 100 μg·mL^−1^ FLAG® Peptide (Sigma‐Aldrich, Saint Louis, MO, USA)] in the capped column at 4 °C with over end mixing. The elution step was repeated with 15 min incubation. Remaining unbound SHBG was eluted with 2 × 2 mL binding buffer.

The anti‐FLAG purified SHBG was additionally purified by anion exchange. The pooled sample was mixed with two volumes of dilution buffer (50 mm Tris‐HCl, 2.5 mm CaCl_2_, pH 7.5), to lower the salt concentration, and loaded on a Mono Q HR 5/5 column (Pharmacia Biotech, Uppsala, Sweden) pre‐equilibrated in buffer A (50 mm Tris‐HCl, 2.5 mm CaCl_2_, 50 mm NaCl, pH 7.5). The column was washed with 10 column volumes of buffer A, whereafter proteins were eluted by applying a linear salt gradient from 0% to 100% buffer B (50 mm Tris‐HCl, pH 7.5, 500 mm NaCl, 2.5 mm CaCl_2_) over 50 column volumes at a flow rate of 0.5 mL·min^−1^. 1 mL fractions were collected and those containing SHBG were combined.

The identity, purity, and integrity of the SHBG proteins were confirmed by SDS/PAGE, western blotting, and MALDI‐ToF MS. The purified protein yield from three independent culture batches was determined using absorption at 280 nm (extinction coefficients for the recombinant ursine and human SHBG are 47230 M^−1^·cm^−1^ and 58230 M^−1^·cm^−1^, respectively).

### Test expression of recombinant SHBG in ExpiSf cells supplemented with DHT

Test expressions were performed in 4 mL suspension cultures in 6‐well plates (CELLSTAR®, Greiner Bio‐One GmbH, Kremsmünster, Austria). ExpiSf cells were seeded, infected, and harvested as previously described, except that DHT dissolved in absolute ethanol was supplemented to the cultures at the time of infection, resulting in final DHT concentrations of 0 and 10 µm, and an ethanol concentration of 1% in the cultures (ethanol in this concentration did not affect the morphology of the cells).

### Western blot analysis

The proteins were blotted from an SDS/PAGE (4–20%, except for native PAGE where a 10% gel was applied) onto a nitrocellulose membrane using a Trans‐Blot® Turbo™ Transfer System (Bio‐Rad Laboratories, Singapore).

Recombinant SHBG were immunostained using 10 µg·mL^−1^ murine monoclonal primary antibody to anti‐FLAG® M2 (Eastman Kodak Company, New Haven, CT, USA) and 0.2 µg·mL^−1^ Alexa Fluor® 546 donkey anti‐mouse IgG (H + L) secondary antibody (Invitrogen™, Life Technologies). The Alexa 546 fluorophore was detected with λex/λem 520−545/577–613 nm, while the bands of the molecular weight marker were visualized with λex/λem 625−650/675–725 nm. Protein band intensities on the western‐blotted membranes were quantified using the imagej 1.52a software [47].

For comparison of intracellular and extracellular SHBG, cells and expression medium were separated by centrifugation at 300 ***g*** for 10 min, and the cells were resuspended in PBS corresponding to the original volume. Equal volumes of intra‐ and extracellular samples were loaded on the gel.

For immunostaining of recombinant SHBG in a native western blot, affinity purified rabbit anti‐SHBG LG4 primary antibody (provided by Geoffrey Hammond, The University of British Columbia) and Goat anti‐rabbit IgG (H + L), HRP secondary antibody (Invitrogen™, Life Technologies) was applied and detected by Amersham ECL Prime Western Blotting Detection Reagent (GE Healthcare, Buckinghamshire, UK).

### MALDI‐ToF MS

#### Sample preparation for digested SHBG

Tryptically digested SHBG was analyzed at both reducing and nonreducing conditions and chymotryptically digested SHBG at reducing conditions.

Purified SHBG was mixed 1 : 1 (v/v) with digestion buffer [2% (w/v) sodium deoxycholate in 100 mm triethylammonium bicarbonate] and incubated 10 min at 99 °C. For the samples to be reduced, tris(2‐carboxyethyl)phosphine was added in a 1 : 25 (w/w) reagent‐to‐protein ratio after cooling below 37 °C and incubated 30 min at 37 °C, whereafter iodoacetamide was added in a 1 : 10 (w/w) ratio and the sample incubated for 20 min at 37 °C in the dark. Trypsin or chymotrypsin was added in a 1 : 50 (w/w) ratio and the samples incubated overnight at 37 °C.

To precipitate sodium deoxycholate, formic acid was added to a concentration of 2.0% and the sample incubated at room temperature for 5 min. The samples were then centrifuged at 13 000 ***g*** for 20 min at 4 °C. The supernatant was desalted in a column with a C18 disc (Empore™, Oxford, PA, USA) and R2 and R3 Poros beads (Applied Biosystems, Foster City, CA, USA) and eluted with 10 µL 80% acetonitrile and 0.1% formic acid.

A total of 0.5 μL desalted protein [or peptide standard II (Bruker Daltonik, Bremen, Germany)] was deposited on an AnchorChip™ MALDI target plate followed by 0.5 μL DHB matrix (Thermo Fisher) (40 mg·mL^−1^ DHB in 80% acetonitrile and 0.1% formic acid).

#### Sample preparation for intact SHBG

The method applied is described by Signor and Erba [48].

Purified SHBG was desalted in a column with a C8 disc (Empore™) and R2 and R3 reversed phase resin and eluted with 10 µL 80% acetonitrile and 0.1% formic acid. A small amount of saturated α‐CHCA acetone solution (Thermo Fisher) was deposited on an AnchorChip™ MALDI target plate to form a matrix thin layer. 0.5 μL desalted protein [or protein standard II (Bruker Daltonik)] was deposited on the α‐CHCA thin layer followed by 0.5 μL matrix solution: α‐CHCA [20 mg·mL^−1^ in ACN and 5% formic acid (70 : 30, v/v)] mixed in a 1 : 1 ratio (v/v) with DHB [20 mg·mL^−1^ in ACN and 0.1% trifluoroacetic acid (TFA) (70 : 30, v/v)].

MALDI‐ToF MS was performed using a Bruker Daltonics autoFlex TOF/TOF MALDI‐ToF instrument. Spectra were obtained in positive reflector mode for peptides and in positive linear mode for intact protein.

### Size exclusion chromatography with multiangle light scattering analysis

SEC‐MALS was used to evaluate the molecular masses of SHBG. SEC measurements were performed on an Agilent 1260 Infinity HPLC system in‐line with a WYATT 30S5 guard column and a WYATT 30S5 SEC column. A MALS detector DAWN HELEOS 8 (Wyatt Technology, Santa Barbara, CA, USA) was used to obtain protein molecular mass and the refractometer Optilab T‐rEX (Wyatt Technology) to measure the refractive index (dRI). The system was equilibrated overnight in 20 mm Tris‐HCl, 150 mm NaCl, 1 mm CaCl_2_, 1 μm DHT, pH 8.0 (0.22 μm filtered and sonicated). For the ursine measurements, 250 mm NaCl was applied to prevent the protein from sticking to each other. 100 μL sample was injected (~ 10 μg SHBG) and BSA (2 mg·mL^−1^) was used for calibration. The flow rate was 0.5 mL·min^−1^. Data analysis was performed using the astra® software (Wyatt Technology).

### Thermal unfolding profiles

Five hundred nanomolar purified ursine and human SHBG was equilibrated with 0 or 50 μm DHT in triplicates. Thermal unfolding profiles from 20.0 to 95.0 °C and a temperature increase of 1.0 °C·min^−1^ were measured on a Prometheus NT.48 system (NanoTemper Technologies, München, Germany) using high‐sensitivity capillaries. The unfolding temperatures were determined as the peak maximum of the first derivative of the fluorescent ratio 350 nm/330 nm as a function of the temperature.

### Steroid binding assays

The binding affinities and relative binding affinities (RBA) of ursine and human SHBG were measured by steroid ligand saturation analysis according to the method by Hammond and Lähteenmäki [39].

The optimal dextran‐coated charcoal (DCC) exposure time was determined to 10 min by time‐course experiments, which ensured efficient adsorption of the majority of the nonbound [1,2‐^3^H]5α‐dihydrotestosterone ([^3^H]DHT) (specific activity 60 Ci·mmol^−1^, American Radiolabelled Chemicals) with minimal dissociation of bound complex (Fig. S9A). The percentage dissociation of bound complex during the 10 min DCC exposure was determined by extrapolation of the counts from specifically bound [^3^H]DHT to SHBG to zero time and used to calculate the dissociation rate of the complex (Fig. S9B,C). After 10‐min DCC exposure, approximately 23% of the ursine SHBG‐DHT complex and 6% of the human SHBG‐DHT complex had irreversibly dissociated. Consequently, a correction factor of 1.23 for ursine SHBG and 1.06 for human SHBG was applied in the calculations of SHBG binding capacity to account for dissociation during the DCC treatment.

#### Determination of SHBG‐binding affinities to DHT

The steroid‐binding affinities of purified recombinant ursine and human SHBG to [^3^H]DHT were determined by equilibration with [^3^H]DHT in 7‐step twofold dilutions in the presence or absence of 100‐fold molar excess of unlabeled DHT (Steraloids, Inc., Newport, RI, USA) and removal of unbound steroid by 10‐min DCC exposure. For Scatchard analysis, bound steroid over free steroid was plotted as a function of bound steroid. The free steroid count is calculated by subtracting bound steroid count from the total [^3^H]DHT count added. Linear regression analysis was performed to obtain the dissociation constant (*K*_D_), corresponding to the negative reciprocal of the slope.

#### Determination of relative binding affinities

The RBAs were determined in a competitive steroid‐binding assay, by saturating SHBG with [^3^H]DHT (approximately 6 nm) and adding increasing amounts of the following unlabeled steroid competitors: estradiol, testosterone, androstenediol, 3β‐androstanediol, dehydroepiandrosterone (Aldrich Chemical Company, Inc., Milwaukee, WI, USA), androstenedione (Sigma Chemical Co., St. Louis, MO, USA), progesterone, 11‐deoxycorticosterone, 11‐deoxycortisol, and corticosterone. Additionally, the RBA for estradiol was assessed in the presence and absence of 1 mm Zn^2+^. Steroids were purchased from Steraloids, Inc., unless stated otherwise. RBA were calculated in percentage from the IC_50_ values, that is, the concentrations of steroid that result in 50% displacement of the bound [^3^H]DHT relative to the concentration required of unlabeled DHT.

*K*_D_ values of the steroids were calculated from the IC_50_ values, according to the Cheng–Prusoff equation [41]:

$$K_{D(\text{competitor})}=\frac{{\text{IC}}_{50}}{1+\frac{\left[{[}^{3}H]{\text{DHT}}_{\text{free}}\right]}{K_{D({[}^{3}H]\text{DHT})}}}.$$

## Conflict of interest

The authors declare no conflict of interest.

## Author contributions

AMF wrote the first manuscript draft. AMF, PR, GLH, MB, and MTO designed the experiments. AMF, JNCT, and PR performed the experimental work and data analysis. AMF, MB, GLH, and MTO were in charge of data interpretation. MB, GLH, OF, and MTO revised the manuscript. All authors read and approved the final manuscript.

## Supporting information


**Fig. S1.** Effect of DHT supplementation on the SHBG yield.
**Fig. S2.** Sequence coverage of SHBG in MALDI‐ToF MS.
**Fig. S3.** Identification of the signal peptide cleavage site of ursine SHBG.
**Fig. S4.** Identification of disulfide bond in human SHBG.
**Fig. S5.** Native western blot of SHBG.
**Fig. S6.** Chromatograms from SEC‐MALS.
**Fig. S7.** Determination of SHBG molecular mass by MALDI‐ToF MS.
**Fig. S8.** Saturation curve of DHT binding to SHBG.
**Fig. S9.** Time‐course experiments during DCC exposure.
**Table S1.** Primer sequences used for cloning.

## Data Availability

The data that support the findings of this study are available from the corresponding author [mto@bio.aau.dk] upon reasonable request.

## References

[1] Jänne M , Deol HK , Power SGA , Yee S , Hammond GL . Human sex hormone‐binding globulin gene expression in transgenic mice. Mol Endocrinol. 1998;12:123–36. 9440816 10.1210/mend.12.1.0050

[2] Siiteri PK , Murai JT , Hammond GL , Nisker JA , Raymoure WJ , Kuhn RW . The serum transport of steroid hormones. Recent Prog Horm Res. 1982;38:457–510. 6750727 10.1016/b978-0-12-571138-8.50016-0

[3] Laurent MR , Hammond GL , Blokland M , Jardí F , Antonio L , Dubois V , et al. Sex hormone‐binding globulin regulation of androgen bioactivity in vivo: validation of the free hormone hypothesis. Sci Rep. 2016;6:35539. 27748448 10.1038/srep35539 PMC5066276

[4] Mendel CM . The free hormone hypothesis: a physiologically based mathematical model. Endocr Rev. 1989;10:232–74. 2673754 10.1210/edrv-10-3-232

[5] Welinder KG , Hansen R , Overgaard MT , Brohus M , Sønderkær M , von Bergen M , et al. Biochemical foundations of health and energy conservation in hibernating free‐ranging subadult brown bear *Ursus arctos* . J Biol Chem. 2016;291:22509–23. 27609515 10.1074/jbc.M116.742916 PMC5077189

[6] Nelson RA . Winter sleep in the black bear. A physiologic and metabolic marvel. Mayo Clin Proc. 1973;48:733–7. 4745546

[7] Berg von Linde M , Arevström L , Fröbert O . Insights from the Den: how hibernating bears may help us understand and treat human disease. Clin Transl Sci. 2015;8:601–5. 26083277 10.1111/cts.12279 PMC5351099

[8] Dittmer DK , Teasell R . Complications of immobilization and bed rest. Part 1: musculoskeletal and cardiovascular complications. Can Fam Physician. 1993;39:1428–37. 8324411 PMC2379624

[9] Teasell R , Dittmer DK . Complications of immobilization and bed rest. Part 2: Other complications. Can Fam Physician. 1993;39:1440–6. 8324412 PMC2379609

[10] Toljan K , Grgić F , Baldani DP , Jurković I , Goldstajn MS . Sex Hormone Binding Globulin (SHBG) as a marker of clinical disorders. Coll Antropol. 2016;40:211–8. 29139641

[11] Vos MJ , Mijnhout GS , Rondeel JMM , Baron W , Groeneveld PHP . Sex hormone binding globulin deficiency due to a homozygous missense mutation. J Clin Endocrinol Metab. 2014;99:E1798–802. 24937543 10.1210/jc.2014-2055

[12] Bocchinfuso WP , Warmels‐Rodenhiser S , Hammond GL . Expression and differential glycosylation of human sex hormone‐binding globulin by mammalian cell lines. Mol Endocrinol. 1991;5:1723–9. 1723489 10.1210/mend-5-11-1723

[13] Hagen FS , Arguelles C , Sui L , Zhang W , Seidel PR , Conroy SC , et al. Mammalian expression of the human sex steroid‐binding protein of plasma (SBP or SHBG) and testis (ABP) characterization of the recombinant protein. FEBS Lett. 1992;299:23–7. 1544469 10.1016/0014-5793(92)80091-t

[14] Sui LM , Wong C , Petra PH . Over‐expression of human sex steroid‐binding protein (hSBP/hABP or hSHBG) in insect cells infected with a recombinant baculovirus. Characterization of the recombinant protein and comparison to the plasma protein. J Steroid Biochem Mol Biol. 1995;52:173–9. 7532988 10.1016/0960-0760(94)00156-g

[15] Hildebrand C , Bocchinfuso WP , Dales D , Hammond GL . Resolution of the steroid‐binding and dimerization domains of human sex hormone‐binding globulin by expression in *Escherichia coli* . Biochemistry. 1995;34:3231–8. 7880817 10.1021/bi00010a012

[16] Grishkovskaya I , Sklenar G , Avvakumov GV , Dales D , Behlke J , Hammond GL , et al. Crystallization of the N‐terminal domain of human sex hormone‐binding globulin, the major sex steroid carrier in blood. Acta Crystallogr D Biol Crystallogr. 1999;55:2053–5. 10666590 10.1107/s0907444999012883

[17] Sui LM , Lennon J , Ma C , McCann I , Woo I , Pétra PH . Heterologous expression of wild type and deglycosylated human sex steroid‐binding protein (SBP or SHBG) in the yeast, *Pichia pastoris*. Characterization of the recombinant proteins. J Steroid Biochem Mol Biol. 1999;68:119–27. 10369409 10.1016/s0960-0760(99)00024-2

[18] Hilpert J , Vorum H , Burmeister R , Spoelgen R , Grishkovskaya I , Misselwitz R , et al. Efficient eukaryotic expression system for authentic human sex hormone‐binding globulin. Biochem J. 2001;360:609–15. 11736650 10.1042/0264-6021:3600609 PMC1222263

[19] Walsh KA , Titani K , Takio K , Kumar S , Hayes R , Petra PH . Amino acid sequence of the sex steroid binding protein of human blood plasma. Biochemistry. 1986;25:7584–90. 3542030 10.1021/bi00371a048

[20] Petra PH , Kumar S , Hayes R , Ericsson LH , Titani K . Molecular organization of the sex steroid‐binding protein (SBP) of human plasma. J Steroid Biochem. 1986;24:45–9. 3702428 10.1016/0022-4731(86)90030-0

[21] Petra PH , Stanczyk FZ , Senear DF , Namkung PC , Novy MJ , Ross JB , et al. Current status of the molecular structure and function of the plasma sex steroid‐binding protein (SBP). J Steroid Biochem. 1983;19:699–706. 6684195 10.1016/0022-4731(83)90238-8

[22] Grishkovskaya I , Avvakumov GV , Sklenar G , Dales D , Hammond GL , Muller YA . Crystal structure of human sex hormone‐binding globulin: steroid transport by a laminin G‐like domain. EMBO J. 2000;19:504–12. 10675319 10.1093/emboj/19.4.504 PMC305588

[23] Strel'chyonok OA , Avvakumov GV , Survilo LI . A recognition system for sex‐hormone‐binding protein‐estradiol complex in human decidual endometrium plasma membranes. Biochim Biophys Acta. 1984;802:459–66. 6542426 10.1016/0304-4165(84)90365-9

[24] Rosner W , Hryb DJ , Kahn SM , Nakhla AM , Romas NA . Interactions of sex hormone‐binding globulin with target cells. Mol Cell Endocrinol. 2010;316:79–85. 19698759 10.1016/j.mce.2009.08.009

[25] Khan MS , Hryb DJ , Hashim GA , Romas NA , Rosner W . Delineation and synthesis of the membrane receptor‐binding domain of sex hormone‐binding globulin. J Biol Chem. 1990;265:18362–5. 2170408

[26] Grishkovskaya I , Avvakumov GV , Hammond GL , Catalano MG , Muller YA . Steroid ligands bind human sex hormone‐binding globulin in specific orientations and produce distinct changes in protein conformation. J Biol Chem. 2002;277:32086–93. 12065592 10.1074/jbc.M203999200

[27] Sumer‐Bayraktar Z , Nguyen‐Khuong T , Jayo R , Chen DDY , Ali S , Packer NH , et al. Micro‐ and macroheterogeneity of N‐glycosylation yields size and charge isoforms of human sex hormone binding globulin circulating in serum. Proteomics. 2012;12:3315–27. 23001782 10.1002/pmic.201200354

[28] Avvakumov GV , Matveentseva IV , Akhrem LV , Strel'chyonok OA , Akhrem AA . Study of the carbohydrate moiety of human serum sex hormone‐binding globulin. Biochim Biophys Acta Gen Sub. 1983;760:104–10. 10.1016/0304-4165(83)90130-7 6684483

[29] Cousin P , Déchaud H , Grenot C , Lejeune H , Hammond GL , Pugeat M . Influence of glycosylation on the clearance of recombinant human sex hormone‐binding globulin from rabbit blood. J Steroid Biochem Mol Biol. 1999;70:115–21. 10622399 10.1016/s0960-0760(99)00101-6

[30] Avvakumov GV , Muller YA , Hammond GL . Steroid‐binding specificity of human sex hormone‐binding globulin is influenced by occupancy of a zinc‐binding site. J Biol Chem. 2000;275:25920–5. 10859323 10.1074/jbc.M004484200

[31] Avvakumov GV , Cherkasov A , Muller YA , Hammond GL . Structural analyses of sex hormone‐binding globulin reveal novel ligands and function. Mol Cell Endocrinol. 2010;316:13–23. 19748550 10.1016/j.mce.2009.09.005

[32] Olczak M , Olczak T . Comparison of different signal peptides for protein secretion in nonlytic insect cell system. Anal Biochem. 2006;359:45–53. 17046707 10.1016/j.ab.2006.09.003

[33] Hammond GL , Robinson PA , Sugino H , Ward DN , Finne J . Physicochemical characteristics of human sex hormone binding globulin: evidence for two identical subunits. J Steroid Biochem. 1986;24:815–24. 3702459 10.1016/0022-4731(86)90442-5

[34] Nielsen H . Predicting secretory proteins with SignalP. Methods Mol Biol. 2017;1611:59–73. 28451972 10.1007/978-1-4939-7015-5_6

[35] Tessier DC , Thomas DY , Khouri HE , Laliberté F , Vernet T . Enhanced secretion from insect cells of a foreign protein fused to the honeybee melittin signal peptide. Gene. 1991;98:177–83. 2016060 10.1016/0378-1119(91)90171-7

[36] Futatsumori‐Sugai M , Tsumoto K . Signal peptide design for improving recombinant protein secretion in the baculovirus expression vector system. Biochem Biophys Res Commun. 2010;391:931–5. 19962965 10.1016/j.bbrc.2009.11.167

[37] Nilsson I , Heijne GV . A signal peptide with a proline next to the cleavage site inhibits leader peptidase when present in a sec‐independent protein. FEBS Lett. 1992;299:243–6. 1544500 10.1016/0014-5793(92)80124-y

[38] Hammond GL , Miguel‐Queralt S , Yalcinkaya TM , Underhill C , Place NJ , Glickman SE , et al. Phylogenetic comparisons implicate sex hormone‐binding globulin in “masculinization” of the female spotted hyena (*Crocuta crocuta*). Endocrinology. 2012;153:1435–43. 22253421 10.1210/en.2011-1837 PMC3281530

[39] Hammond GL , Lähteenmäki PLA . A versatile method for the determination of serum cortisol binding globulin and sex hormone binding globulin binding capacities. Clin Chim Acta. 1983;132:101–10. 6193907 10.1016/0009-8981(83)90237-1

[40] Renoir JM , Mercier‐Bodard C , Baulieu EE . Hormonal and immunological aspects of sex steroid‐binding plasma protein of primates. J Reprod Fertil Suppl. 1980;28:113–9. 6934306

[41] Cheng Y , Prusoff WH . Relationship between the inhibition constant (K1) and the concentration of inhibitor which causes 50 per cent inhibition (I50) of an enzymatic reaction. Biochem Pharmacol. 1973;22:3099–108. 4202581 10.1016/0006-2952(73)90196-2

[42] Pétra PH , Woodcock KT , Orr WR , Nguyen DK , Sui LM . The sex steroid binding protein (SBP or SHBG) of human plasma: identification of Tyr‐57 and Met‐107 in the steroid binding site. J Steroid Biochem Mol Biol. 2000;75:139–45. 11226830 10.1016/s0960-0760(00)00169-2

[43] Bocchinfuso WP , Warmels‐Rodenhiser S , Hammond GL . Structure/function analyses of human sex hormone‐binding globulin by site‐directed mutagenesis. FEBS Lett. 1992;301:227–30. 1568485 10.1016/0014-5793(92)81253-i

[44] Hammond GL , Avvakumov GV , Muller YA . Structure/function analyses of human sex hormone‐binding globulin: effects of zinc on steroid‐binding specificity. J Steroid Biochem Mol Biol. 2003;85:195–200. 12943704 10.1016/s0960-0760(03)00195-x

[45] Waterhouse A , Bertoni M , Bienert S , Studer G , Tauriello G , Gumienny R , et al. SWISS‐MODEL: homology modelling of protein structures and complexes. Nucleic Acids Res. 2018;46:W296–303. 29788355 10.1093/nar/gky427 PMC6030848

[46] Schrodinger L . The PyMOL Molecular Graphics System, Version 2.4. 2010.

[47] Schneider CA , Rasband WS , Eliceiri KW . NIH image to ImageJ: 25 years of image analysis. Nat Methods. 2012;9:671–5. 22930834 10.1038/nmeth.2089 PMC5554542

[48] Signor L , Boeri Erba E . Matrix‐assisted Laser Desorption/Ionization Time of Flight (MALDI‐TOF) mass spectrometric analysis of intact proteins larger than 100 kDa. J vis Exp. 2013;79:e50635. 10.3791/50635 PMC3857990 24056304

