# Supplementary material for: Characterization and comparison of recombinant full‐length ursine and human sex hormone‐binding globulin
Source: FEBS Open Bio. 2021 Dec 13;12(2):362–78. doi: 10.1002/2211-5463.13341 (PMC8804615; doi:10.1002/2211-5463.13341)
Supplement: Supplementary file 1 — Fig. S1. Effect of DHT supplementation on the SHBG yield. Fig. S2. Sequence coverage of SHBG in MALDI‐ToF MS. Fig. S3. Identification of the signal peptide cleavage site of ursine SHBG. Fig. S4. Identification of disulfide bond in human SHBG. Fig. S5. Native western blot of SHBG. Fig. S6. Chromatograms from SEC‐MALS. Fig. S7. Determination of SHBG molecular mass by MALDI‐ToF MS. Fig. S8. Saturation curve of DHT binding to SHBG. Fig. S9. Time‐course experiments during DCC exposure. Table S1. Primer sequences used for cloning. [file FEB4-12-362-s001.docx]

## Supporting Figures

**
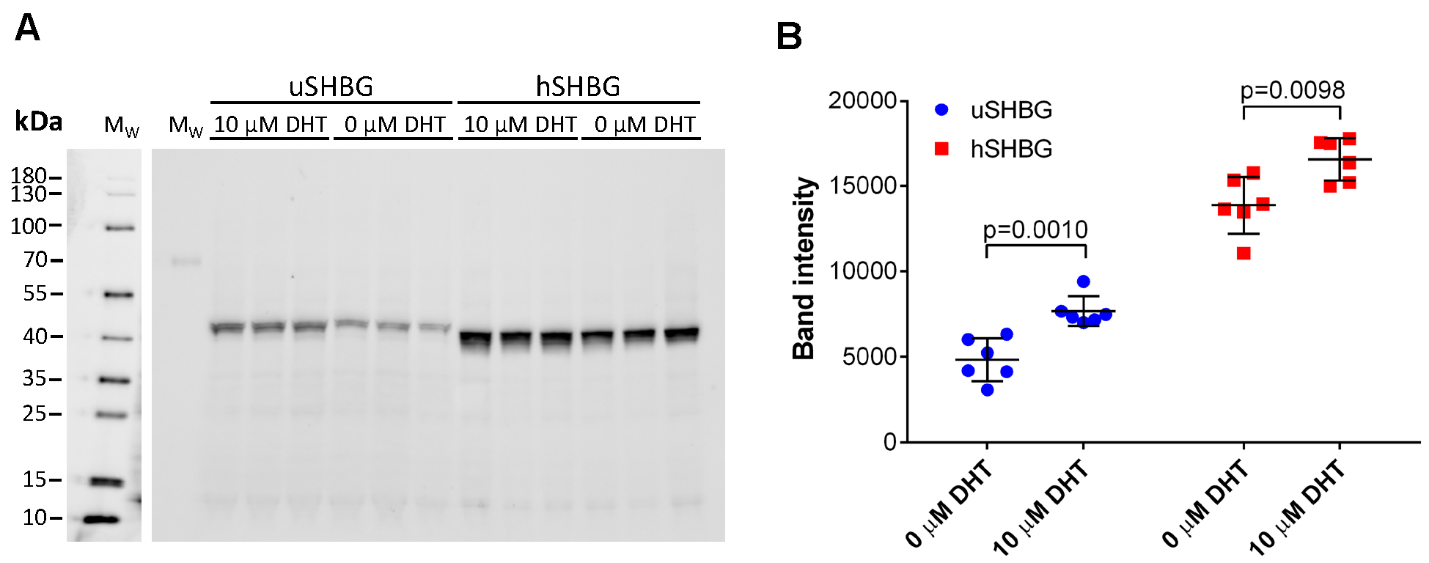
**

**Figure S1:** **Effect of DHT supplementation on the SHBG yield.** A) Triplicate expression of ursine (uSHBG) and human SHBG (hSHBG) in ExpiSf cells supplemented with 0 µM and 10 µM DHT to the expression medium at the time of infection to stabilize SHBG. Ursine samples are loaded in 3x higher volume than the human. B) Band intensities of SHBG from two triplicate test expressions quantified in ImageJ. Means and standard deviations are indicated. P-values were determined from an unpaired student’s t-test.


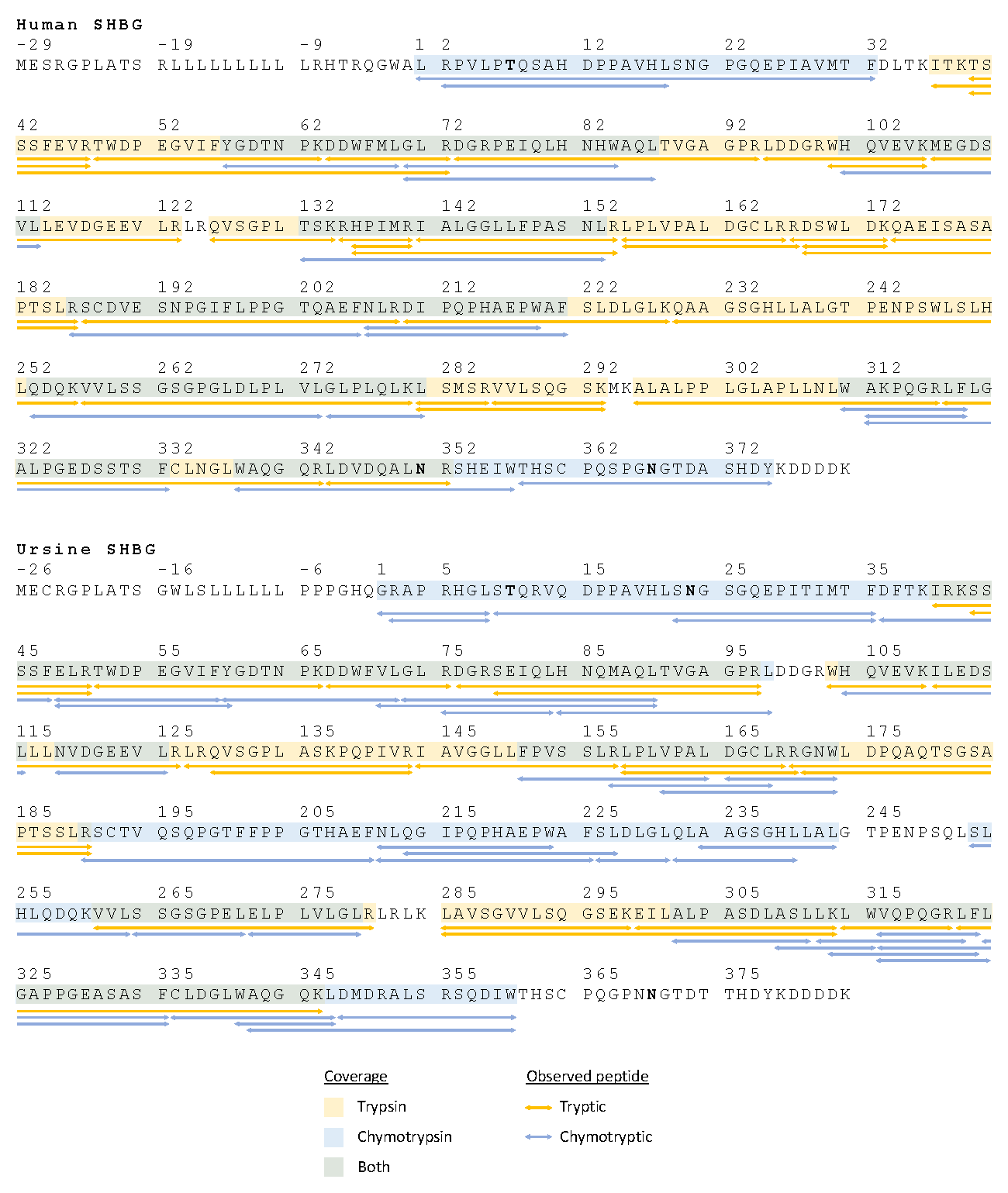


**Figure S2:** **Sequence coverage of SHBG in MALDI-ToF MS.** Sequence coverage in MALDI-ToF MS of tryptically (yellow) and chymotryptically (blue) digested recombinant human and ursine SHBG with a C-terminal FLAG-tag. Parts of the sequence covered by both tryptically and chymotryptically digested SHBG is marked with green.


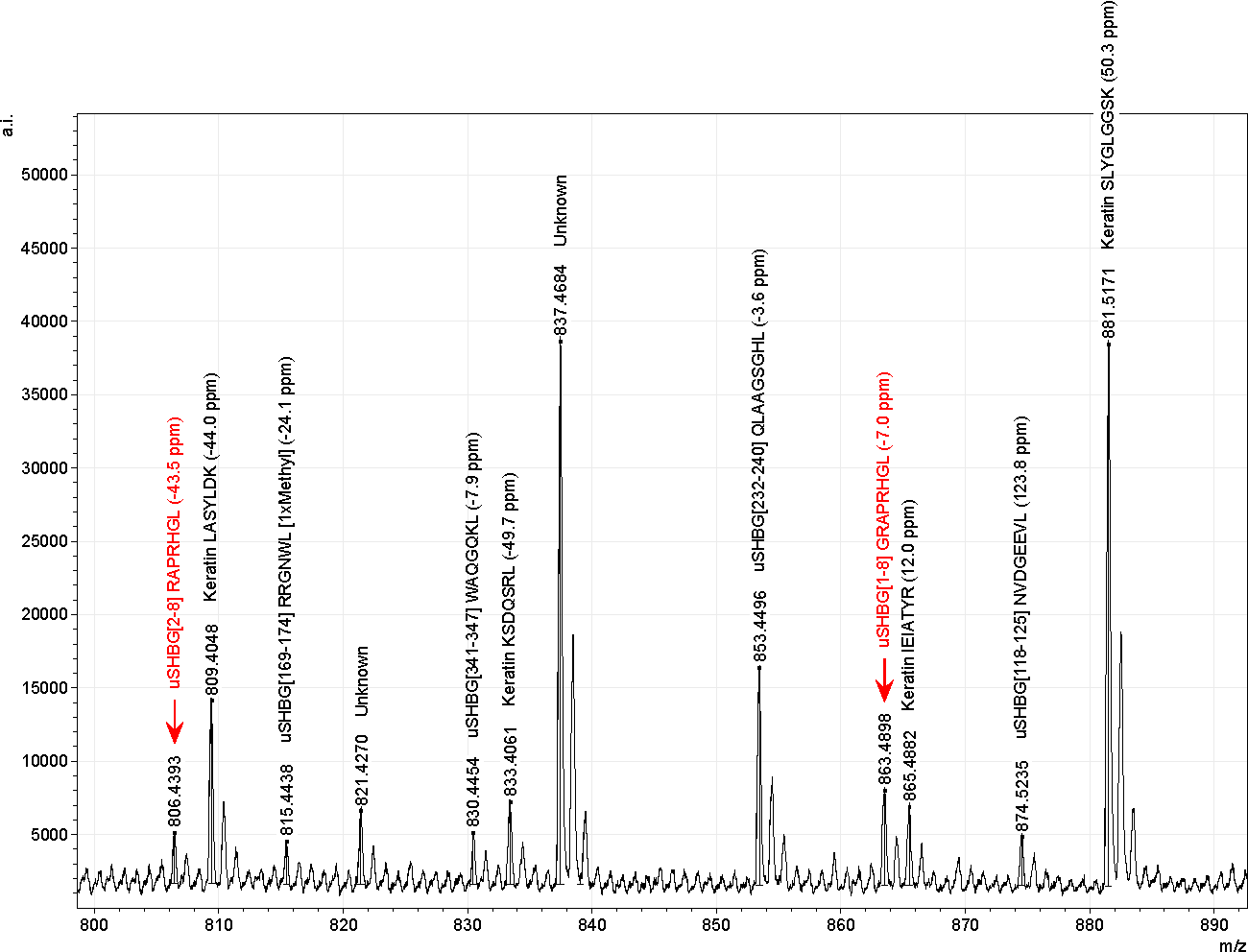


**Figure S3:** **Identification of the signal peptide cleavage site of ursine SHBG.** Section of the mass spectrum from MALDI-ToF MS analysis of ursine SHBG digested with chymotrypsin showing the peaks from the N-terminal semi-chymotryptic peptides where the signal peptide has been cleaved between position 26 and 27 (863.4898 Da – GRAPRHGL) and between position 27 and 28 (806.4220 Da – RAPRHGL), respectively.


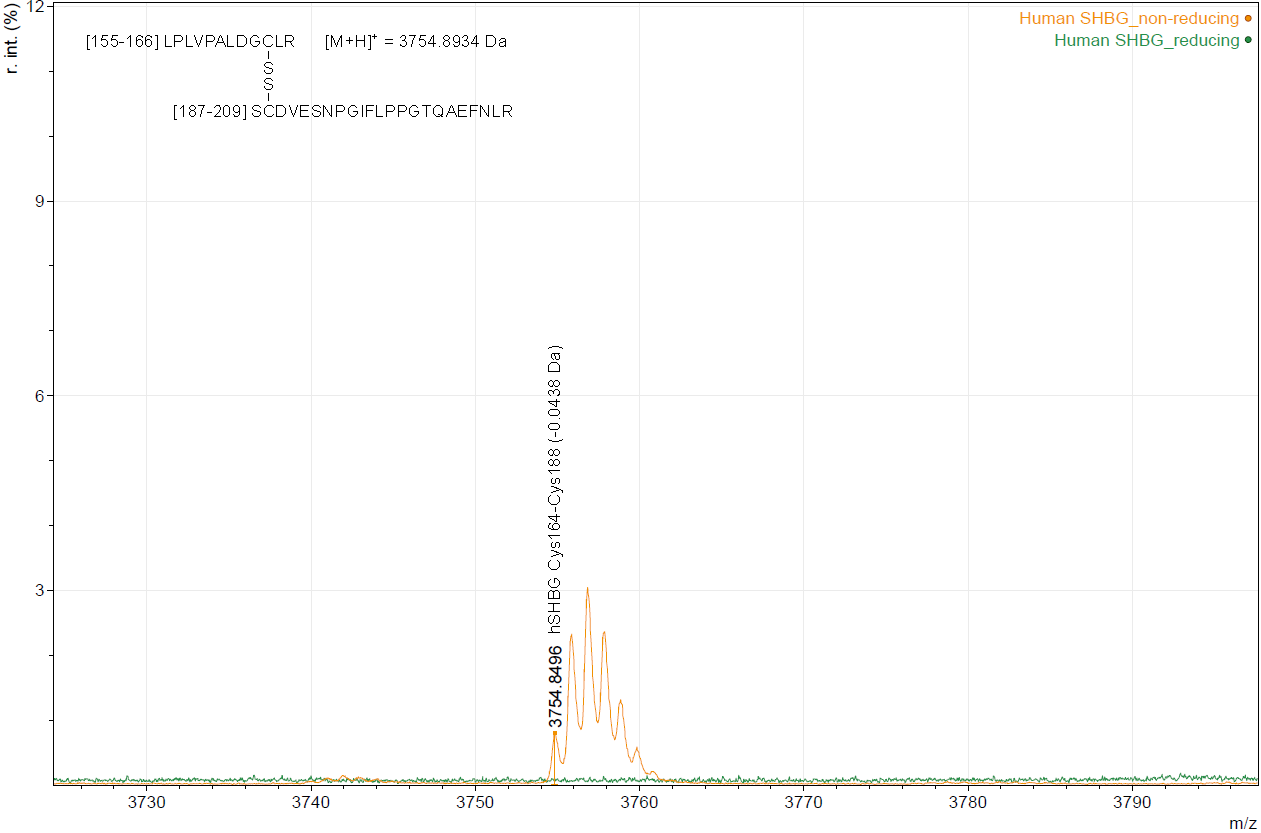


**Figure S4: Identification of disulfide bond in human SHBG.** A peak with a mass corresponding to the tryptic dipeptide with Cys164-Cys188 from human SHBG is observed in MALDI-ToF MS at non-reducing conditions (orange), while being absent at reducing conditions (green).


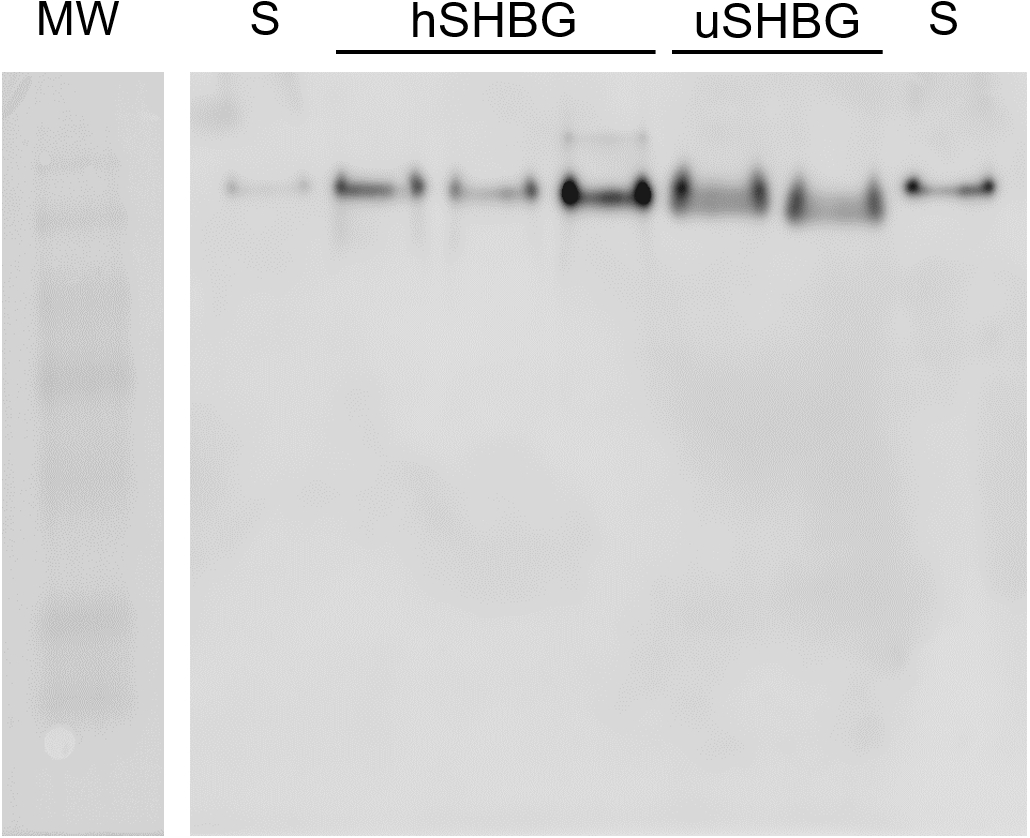


**Figure S5:** **Native western blot of SHBG.** Native western blot of human serum (S) and fractions from AEX of recombinantly expressed human (hSHBG) and ursine SHBG (uSHBG). SHBG was visualized using a SHBG specific antibody and detected with chemiluminescence.


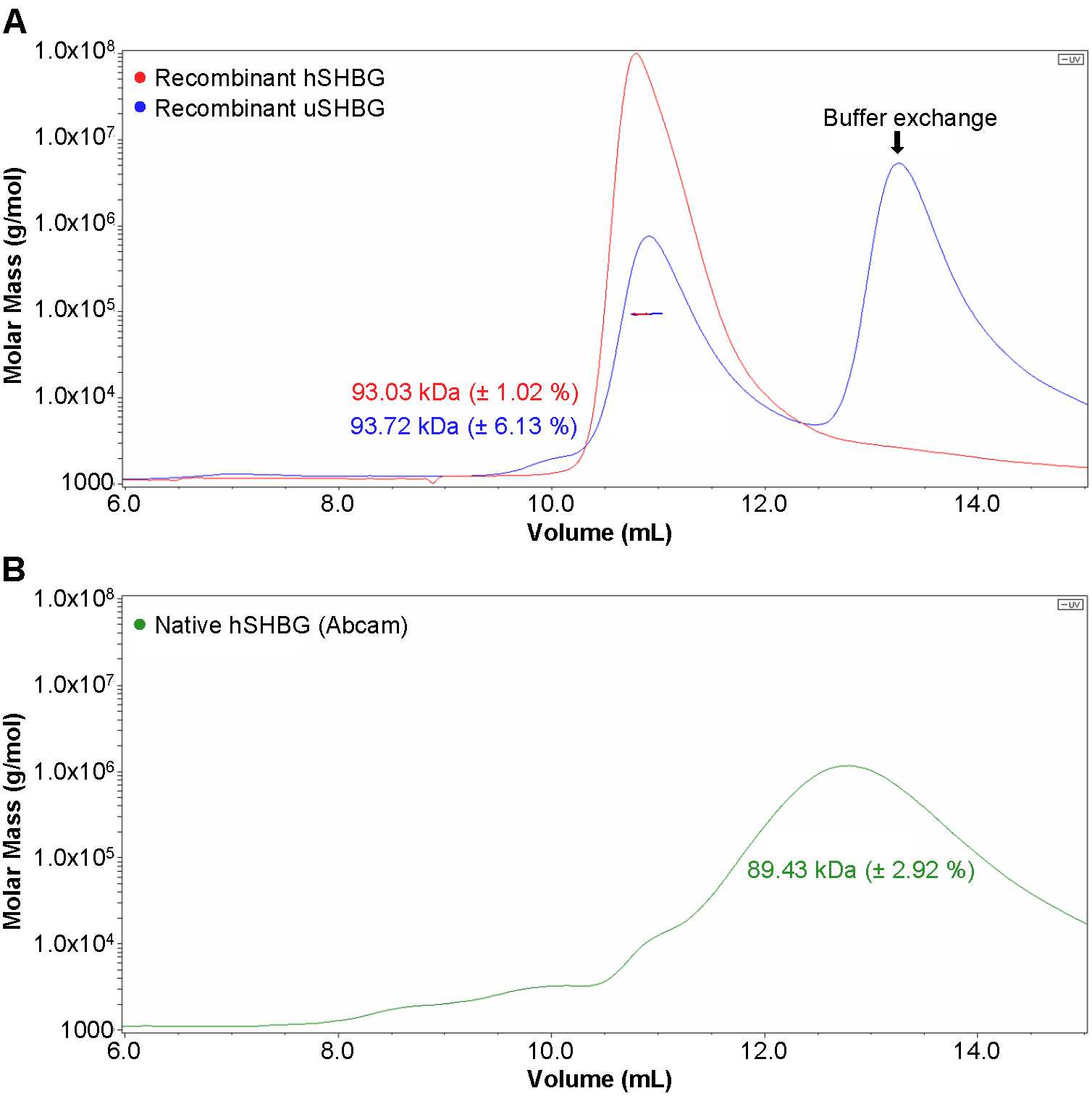


**Figure S6: Chromatograms from SEC-MALS.** Chromatograms from SEC-MALS with UV signal intensities of A) human and ursine recombinant SHBG and B) native human serum SHBG. For the ursine measurements, 250 mM NaCl was applied to prevent the protein from sticking to each other. The increased elution time for native human SHBG is likely due to non-specific interaction with the column matrix. The molecular masses determined in these analyses are stated.


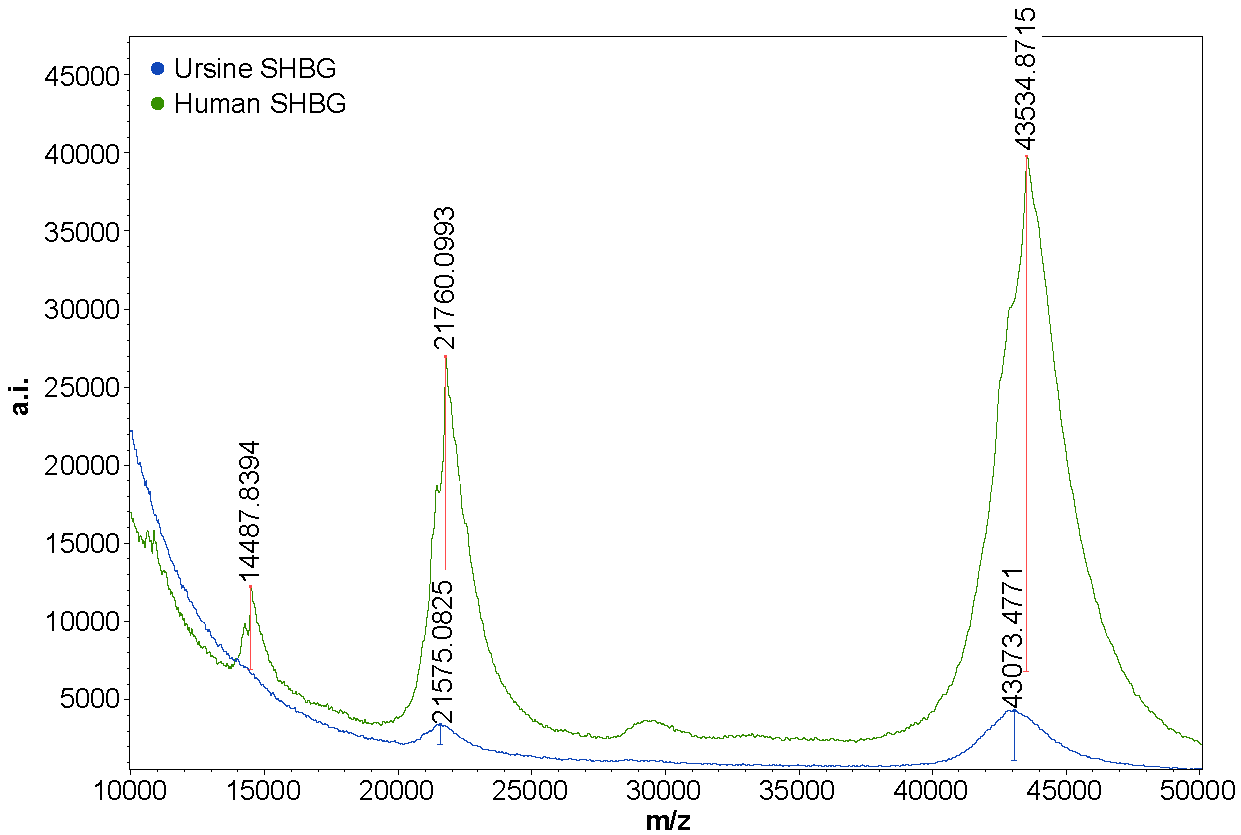


**Figure S7: Determination of SHBG molecular mass by MALDI-ToF MS.** MALDI-ToF MS of intact human and ursine SHBG expressed in ExpiSf cells and purified by α-FLAG affinity chromatography and AEX.


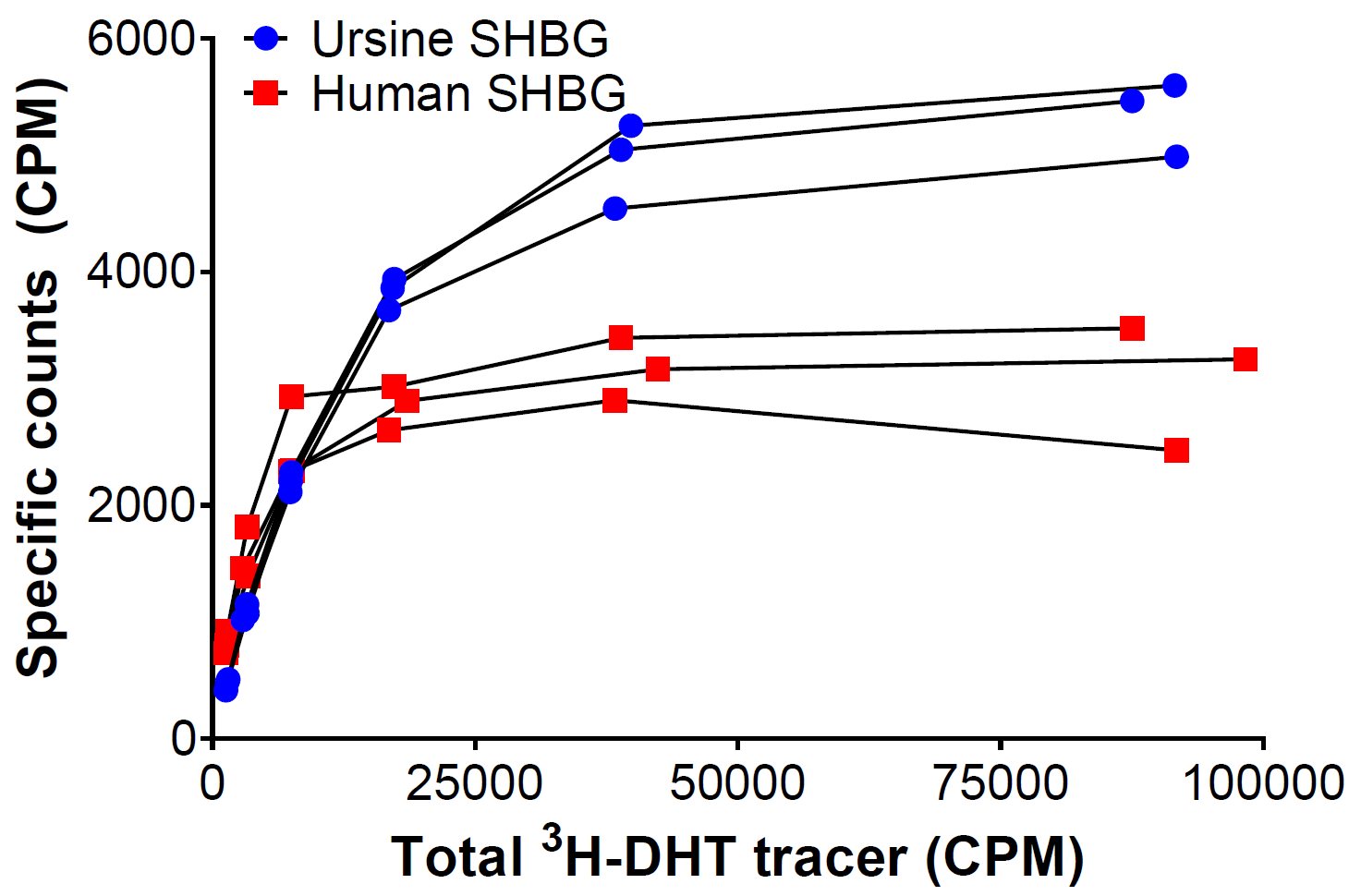


**Figure S8:** **Saturation curve of DHT binding to SHBG.** Saturation curves of specifically bound [^3^H]DHT to SHBG plotted as a function of total [^3^H]DHT added. Based on these results, ~6 nM [^3^H]DHT corresponding to ~50,000 CPM was determined to be sufficient to saturate the applied amount of SHBG.


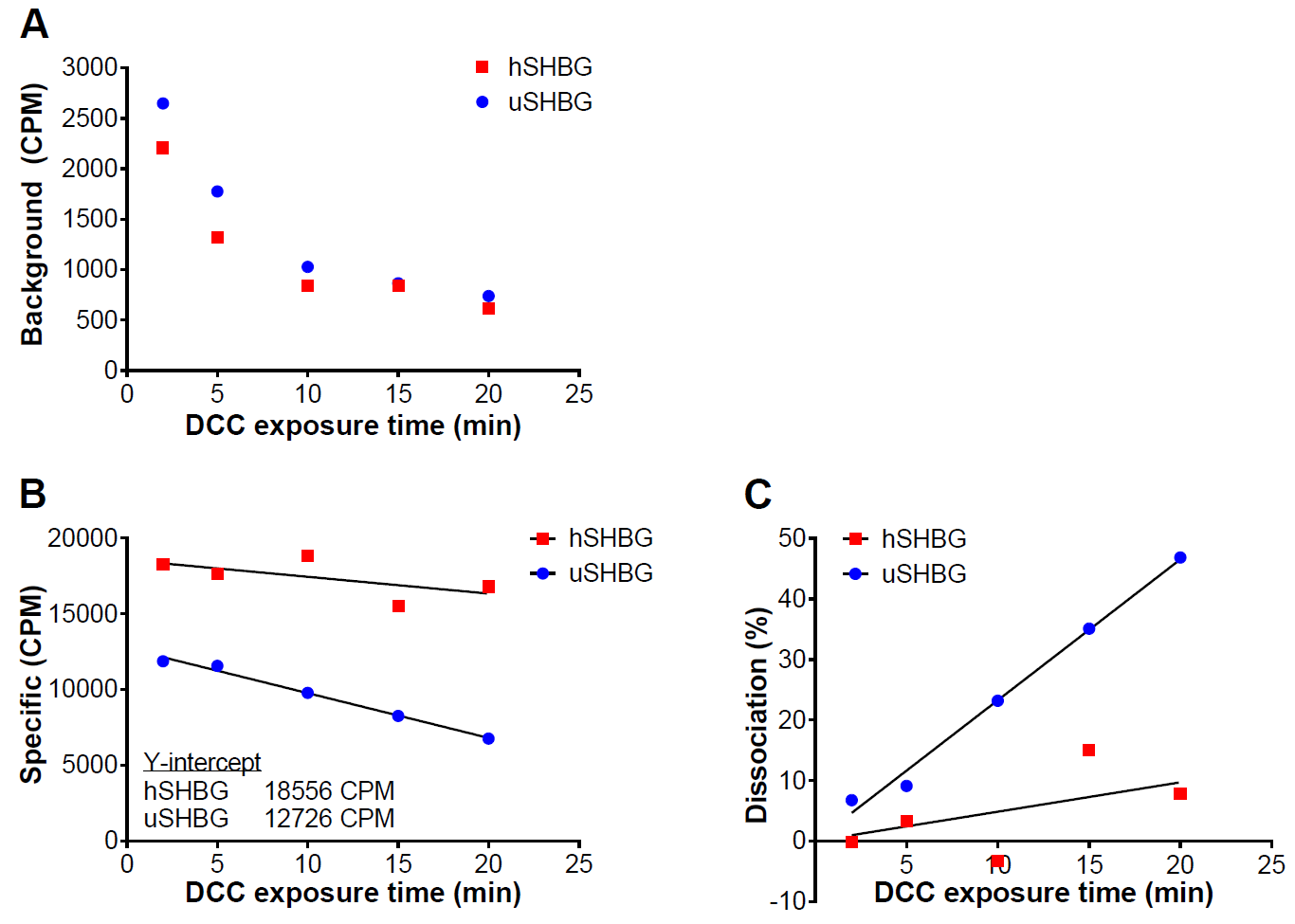


**Figure S9: Time-course experiments during DCC exposure**. A) Background counts plotted as a function of DCC exposure time. B) Counts from specifically bound [^3^H]DHT to SHBG relative to DCC exposure time. C) Dissociation rate of specifically bound [^3^H]DHT to SHBG as a function of DCC exposure time determined by extrapolating the data in figure B to zero time.

## Supporting Tables

**Table S1:** **Primer sequences used for cloning.** List of primer sequences used for amplification of ursine and human shbg cDNA and to substitute the native signal peptide to that of honeybee melittin and for ursine SHBG also to that of human SHBG.

|  | **Primer sequence (5’ → 3’)** | |
| --- | --- | --- |
|  | **Forward** | **Reverse complement** |
| **Human** |  |  |
| Native signal peptide | 5’-TCAGCA^e^-CGGTCCG^b^-ATGGAGAGCAGAGGCCCACT^a^-3’ | 5’-GCACAG^e^-TCTAGA^b^-TTA^c^-CTTGTCGTCATCGTCTTTGTAGTC^d^-ATGGGAAGCGTCAGTGCCA^a^-3’ |
| Honeybee MELT signal peptide | 5’-CACCTA^e^-GCGGCCGC^b^-ATGAAATTCTTAGTCAACGTTGCCCTTGTTTTTATGGTCGTGTACATTTCTTACATCTATGCGGCCCCTGAACCG^f^-CTGAGACCTGTTCTCCCCAC^a^-3’ | -,,- |
| **Ursine** |  |  |
| Native signal peptide | 5’-CACCTA^e^-GCGGCCGC^b^-ATGGAGTGCAGAGGCCCACT^a^-3’ | 5’-GCACAG^e^-TCTAGA^b^-TTA^c^-CTTGTCGTCATCGTCTTTGTAGTC^d^-ATGGGTGGTGTCGGTGCCA^a^-3’ |
| Honeybee MELT signal peptide | 5’-CACCTA^e^-GCGGCCGC^b^-ATGAAATTCTTAGTCAACGTTGCCCTTGTTTTTATGGTCGTGTACATTTCTTACATCTATGCGGCCCCTGAACCG^f^-CGGGCCCCGAGACATGGT^a^-3’ | -,,- |
| Human SHBG signal peptide | 5’-CACCT^e^-GCGGCCGC^b^-ATGGAGAGCAGAGGCCCACTGGCTACCTCGCGCCTGCTGCTGTTGCTGCTGTTGCTACTACTGCGTCACACCCGCCAGGGATGGGCCCT^f^-CGGGCCCCGAGACATGGT^a^-3’ | -,,- |
| ^a^Complementary region, ^b^restriction site, ^c^stop codon, ^d^FLAG-tag, ^e^random bases, ^f^signal peptide. All stop codons are moved to the 5’ end of the reverse primers, just before the restriction site. | | |
